# Intracellular mGluR5 plays a critical role in neuropathic pain

**DOI:** 10.1038/ncomms10604

**Published:** 2016-02-03

**Authors:** Kathleen Vincent, Virginia M. Cornea, Yuh-Jiin I. Jong, André Laferrière, Naresh Kumar, Aiste Mickeviciute, Jollee S. T. Fung, Pouya Bandegi, Alfredo Ribeiro-da-Silva, Karen L. O'Malley, Terence J. Coderre

**Affiliations:** 1 Alan Edwards Centre for Research on Pain, McGill University, 3655 Promenade Sir William Osler, Montreal, Quebec, Canada H3G 1Y6; 2 Department of Anesthesia, McGill University, 3655 Promenade Sir William Osler, Montreal, Quebec, Canada H3G 1Y6; 3 Department of Anatomy & Neurobiology, Washington University School of Medicine, 660 South Euclid Avenue, St Louis, Missouri 63110, USA; 4 Department of Pharmacology & Therapeutics, McGill University, 3655 Promenade Sir William Osler, Montreal, Quebec, Canada H3G 1Y6

## Abstract

Spinal mGluR5 is a key mediator of neuroplasticity underlying persistent pain. Although brain mGluR5 is localized on cell surface and intracellular membranes, neither the presence nor physiological role of spinal intracellular mGluR5 is established. Here we show that in spinal dorsal horn neurons >80% of mGluR5 is intracellular, of which ∼60% is located on nuclear membranes, where activation leads to sustained Ca^2+^ responses. Nerve injury inducing nociceptive hypersensitivity also increases the expression of nuclear mGluR5 and receptor-mediated phosphorylated-ERK1/2, Arc/Arg3.1 and c-*fos*. Spinal blockade of intracellular mGluR5 reduces neuropathic pain behaviours and signalling molecules, whereas blockade of cell-surface mGluR5 has little effect. Decreasing intracellular glutamate via blocking EAAT-3, mimics the effects of intracellular mGluR5 antagonism. These findings show a direct link between an intracellular GPCR and behavioural expression *in vivo*. Blockade of intracellular mGluR5 represents a new strategy for the development of effective therapies for persistent pain.

## Abstract (web summary)

mGluR5 has been shown to play a role in chronic pain regulation. Here, the authors use membrane permeable and non-transported, impermeable mGluR5 antagonists to show that spinal analgesic effects *in vivo* are mediated by intracellular rather than cell surface mGluR5.

## Graphical abstract

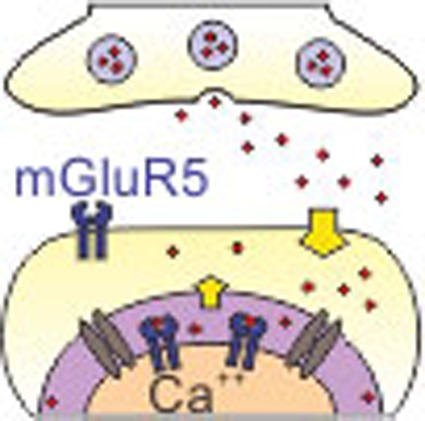

Many G-protein-coupled receptors (GPCRs) are not only expressed at the cell surface but also on various intracellular membranes including the nucleus^1^,^2^,^3^,^4^,^5^,^6^,^7^,^8^,^9^,^10^,^11^,^12^. Although intracellular GPCRs have been shown to play critical roles in gene transcription, ionic homeostasis, cell proliferation, neural circuit remodelling and synaptic plasticity^2^,^5^,^6^,^7^,^12^, the physiological relevance of intracellular receptors in intact organisms remains unknown^1^,^2^,^3^,^4^,^5^,^6^,^7^,^8^,^9^,^10^,^11^,^12^. Metabotropic glutamate 5 receptor (mGluR5) is a GPCR associated with both cell surface and intracellular membranes in striatum, hippocampus and visual cortex, where it couples with G_q/11_/PLC/IP_3_ to release cytoplasmic and nucleoplasmic calcium (Ca^2+^)^7^,^8^,^9^,^10^,^11^. Intracellular mGluR5 is activated following glutamate transport into the cell via excitatory amino-acid transporters (EAATs), or cysteine–glutamate exchangers (xCT), located on cell surface and endoplasmic reticular (ER) membranes^8^. Selective activation of intracellular versus cell-surface mGluR5 triggers unique Ca^2+^ patterns and downstream signalling cascades associated with each receptor pool^7^,^8^,^9^,^10^,^12^.

mGluR5 is abundantly expressed in neurons of the spinal cord dorsal horn (SCDH)^13^,^14^, which serves as the first CNS relay in the transmission of nociceptive information^15^. SCDH mGluR5 plays a key role in glutamate-induced plasticity of pain-related processes, including nociceptive hypersensitivity after nerve injury^16^,^17^,^18^. Specifically, spinal mGluR5 activation induces nociception in normal animals^19^,^20^, while its blockade produces analgesia^16^,^17^,^18^. Despite spinal mGluR5's key role in neuropathic pain^16^,^17^,^18^, it remains unknown whether its effects are due to cell surface or intracellular signalling.

Here we show that mGluR5 and associated effector molecules are increased on SCDH nuclear membranes following spared-nerve injury (SNI), a model of neuropathic pain^21^. Nuclear receptor-associated generation of downstream messengers are also increased. Blockade of spinal intracellular mGluR5 inhibits pain behaviours and mGluR5-linked signalling molecules in nerve-injured rats, whereas blockade of cell surface mGluR5 has little effect. Finally, inhibition of spinal EAAT3 mimics the effects of intracellular mGluR5 antagonism by preventing intracellular uptake of ligand. Our results demonstrate a selective involvement of spinal intracellular mGluR5 in pain processing and provide *in vivo* evidence for a pathophysiological function of a GPCR associated with intracellular membranes.

## Results

### SCDH nuclear mGluR5 activates nuclear Ca^2+^ responses

**Figure 1:**
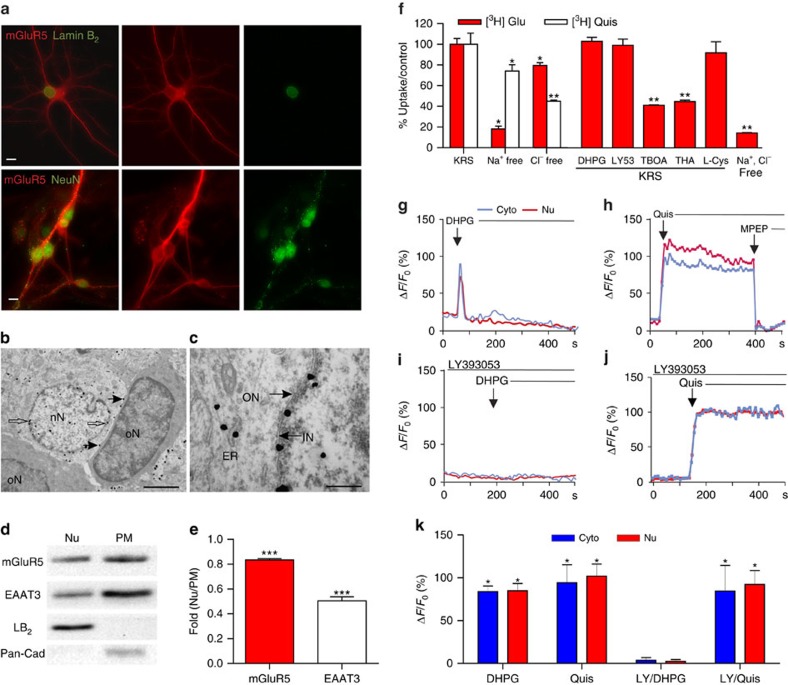
Functional nuclear mGluR5 in SCDH neurons. Fluorescence-microscopy showing (**a**) mGluR5 (red), Lamin-B_2_ (green-upper) or NeuN-IR (green-lower) in cultured rat SCDH neurons. Scale bar, 10 μm. (**b**,**c**) Electron-micrographs showing mGluR5-immunogold in L4–L6 SCDH. Scale bar, (**b**) 2 μm, (**c**) 0.5 μm. mGluR5 is detected in cytoplasm and neuronal nuclei (nN), and on nuclear (white arrows) and plasma (black arrows) membranes, but not glial nuclei (oN, oligodendrocyte nucleus) (**b**). mGluR5 is on inner (IN), and outer (ON), nuclear membranes (black arrows) and on endoplasmic reticular (ER) membranes (**c**). (**d**) Western blots of mGluR5, EAAT3, Lamin-B_2_ (LB_2_), and Pan-cadherin (Pan-Cad) in nuclear (Nu), or plasma membrane (PM) fractions of rat SCDH (L4–L6), quantified in **e**. Data shown represent the mean of three experiments, Student's *t*-test ***P<0.001. (**f**) [^3^H]-glutamate (Glu) or [^3^H]-Quis uptake in cultured rat SCDH neurons with buffer modified as indicated. DHPG (100 μM), LY393053 (LY53: 20 μM), or L-cystine (400 μM) did not block [^3^H]-Glu uptake, whereas TBOA or THA (100 μM) inhibited it ∼60%. Data shown represent the mean of three experiments done in triplicate, Student's *t*-test *P<0.05, ^**^P<0.01 compared to KRS. (**g**–**j**) Representative traces of cytoplasmic (cyto, blue) or nuclear (red) Ca^2+^ responses to DHPG (100 μM **g**,**i**), quisqualate (Quis, 10 μM, **h**,**j**), MPEP (10 μM, **h**) and/or LY393053 (20 μM, **i**,**j**) in cultured rat SCDH neurons. (**k**) Compiled data from maximum response (Δ*F*/*F*_*o*_, %) from *N=*12 identified neurons (**g**); *N*=32 identified neurons (**h**); *N=*25 identified neurons (**i**); *N=*23 identified neurons (**j**); *P<0.05 compared with baseline. All values in figure are expressed as mean +/− s.e.m. KRS, Krebs–Ringer solution.

We used immunocytochemistry in neonatal SCDH cultures, as well as sections and cellular fractions of adult lumbar (L4–L6) tissue to assess the subcellular distribution of mGluR5. We found SCDH cultures expressed mGluR5 on the cell surface, dendrites and on intracellular membranes including the nucleus where mGluR5 colocalized with lamin-B_2_, a nuclear envelope marker (Fig. 1a; upper panels). Detectable mGluR5 staining was seen only on neurons, identified with the neuronal nuclear antigen, NeuN (Fig. 1a; lower panels). Depending on the tissue preparation, mGluR5-positive neurons constituted ∼30% of the cells plated. Electron microscopy was used to assess mGluR5 subcellular localization in adult rat SCDH with pre-embedding, silver-intensified immunogold labelling. mGluR5 was detected on the plasma membrane and intracellularly especially on nuclear membranes (Fig. 1b,c). Nuclear mGluR5 was only detected on SCDH neurons; glial and endothelial cell nuclei were not labelled (Fig. 1b; Supplementary Fig. 1a,b). No mGluR5 labelling occurred in the absence of primary antibody (Supplementary Fig. 1c), and mGluR5 labelling was prevented by preincubation of primary antibody with a specific mGluR5 blocking peptide (Supplementary Fig. 1d). Subfractionation studies showed mGluR5 in both nuclear and plasma membrane fractions, indicated by membrane-specific markers, lamin-B_2_ and pan-cadherin (Pan-Cad), respectively (Fig. 1d). The neuronal sodium-dependent EAAT3 was also found on nuclear and plasma membranes (Fig. 1d). The ratio of nuclear to plasma membrane protein was higher for mGluR5 than for EAAT3 (Fig. 1e). Thus, mGluR5 is highly expressed on intracellular and especially nuclear membranes of SCDH neurons.

Striatal and hippocampal intracellular mGluR5 can be activated by agonist uptake via glutamate transporters/exchangers^7^,^8^,^9^,^10^. Alternatively, intracellular receptors can be modulated by permeable ligands. Permeability can be gauged using published lipophilicity values (Log*P*) where values >2 are considered membrane permeable^22^. Log*P* values indicate that glutamate (−2.7) and the Group 1, mGluR agonists, quisqualate (Log*P*, −3.9) and DHPG (−2.4) are membrane impermeable, as is the Group 1 antagonist, LY393053 (0.6). In contrast, the mGluR5 antagonist 2-methyl-6-(phenylethynyl)-pyridine (MPEP; 3.3) is membrane permeable^7^,^8^,^9^,^10^. To directly test whether sodium- or chloride-dependent processes were involved in glutamate, quisqualate, DHPG or LY393053 uptake in SCDH neurons, cultures were treated with radiolabelled ligand in the presence and absence of transport or exchange inhibitors. Sodium-dependent transporter activity accounted for ∼80% of glutamate, but only ∼25% of quisqualate uptake, whereas chloride-dependent uptake, likely via xCT transport, blocked only ∼20% of glutamate uptake, but ∼50% of quisqualate uptake (Fig. 1f). Sodium- and chloride-free conditions reduced quisqualate and glutamate uptake by >80% (Fig. 1f). DHPG and LY393053 did not compete with glutamate uptake, confirming their designation as impermeable, non-transported ligands (Fig. 1f). Two potent inhibitors of all EAAT subtypes, threo-β-benzyloxyaspartate (TBOA) and threo-β-OH-aspartic acid (THA) blocked about 60% of glutamate uptake, whereas L-cystine, a blocker of the xCT exchanger, had no significant effect (Fig. 1f). Thus, sodium-dependent, EAAT-mediated activity primarily accounts for intracellular glutamate uptake.

Since mGluR5 couples to G_q/11_ and PLC to generate IP_3_-mediated release of Ca^2+^, we used Ca^2+^ imaging to test whether activation of intracellular SCDH mGluR5 is functionally active. SCDH cultures grown on glass coverslips were loaded with the Ca^2+^ indicator, Oregon Green BAPTA-AM, and subsequently treated with agonists and/or antagonists with variable intracellular access. Bath application of the impermeable, non-transported DHPG (100 μM) led to a rapid, transient Ca^2+^ rise (Fig. 1g). In contrast, the impermeable, transported agonist, quisqualate (10 μM), produced a long, sustained rise in Ca^2+^ in both the cytoplasm and the nucleus, which was terminated by addition of the permeable mGluR5 antagonist, MPEP (10 μM; Fig. 1h). Although the impermeable antagonist LY393053 blocked DHPG-mediated Ca^2+^ responses (Fig. 1i), it did not affect those induced by quisqualate (Fig. 1j). These data indicate that functional activity is generated by two separate pools of mGluR5—on the cell surface and on intracellular membranes.

Although surface receptors always contribute to the time course of the Ca^2+^ response, their appearance of doing so varies depending on the scan speed at which images are collected. Because SCDH mGluR5-positive neurons are not as abundant as in striatal or hippocampal cultures, a scan speed of 5.36 s per scan was used here to capture a larger area with more neurons. Thus, compiled data in Fig. 1k reflect group differences in which the contribution of the surface receptor is obscured due to the slow scan speed.

Results from multiple experiments assessing drug-induced changes of both the amplitude of the initial Ca^2+^ peak and the magnitude of the overall Ca^2+^ response revealed that DHPG peak amplitudes (for example, Fig. 1g) were 61.2±3.56% of the LY393053+quisqualate peak amplitudes (Fig. 1j) and that at most the DHPG response constituted ∼10% (9.79±2.27%) of the overall quisqualate response (∼4 min). Thus, during periods of high activity and/or sustained presynaptic glutamate release, intracellular uptake of glutamate can lead to a Ca^2+^ response that is not only spatially and temporally unique, but also approximately nine times larger than a surface response.

**Figure 2:**
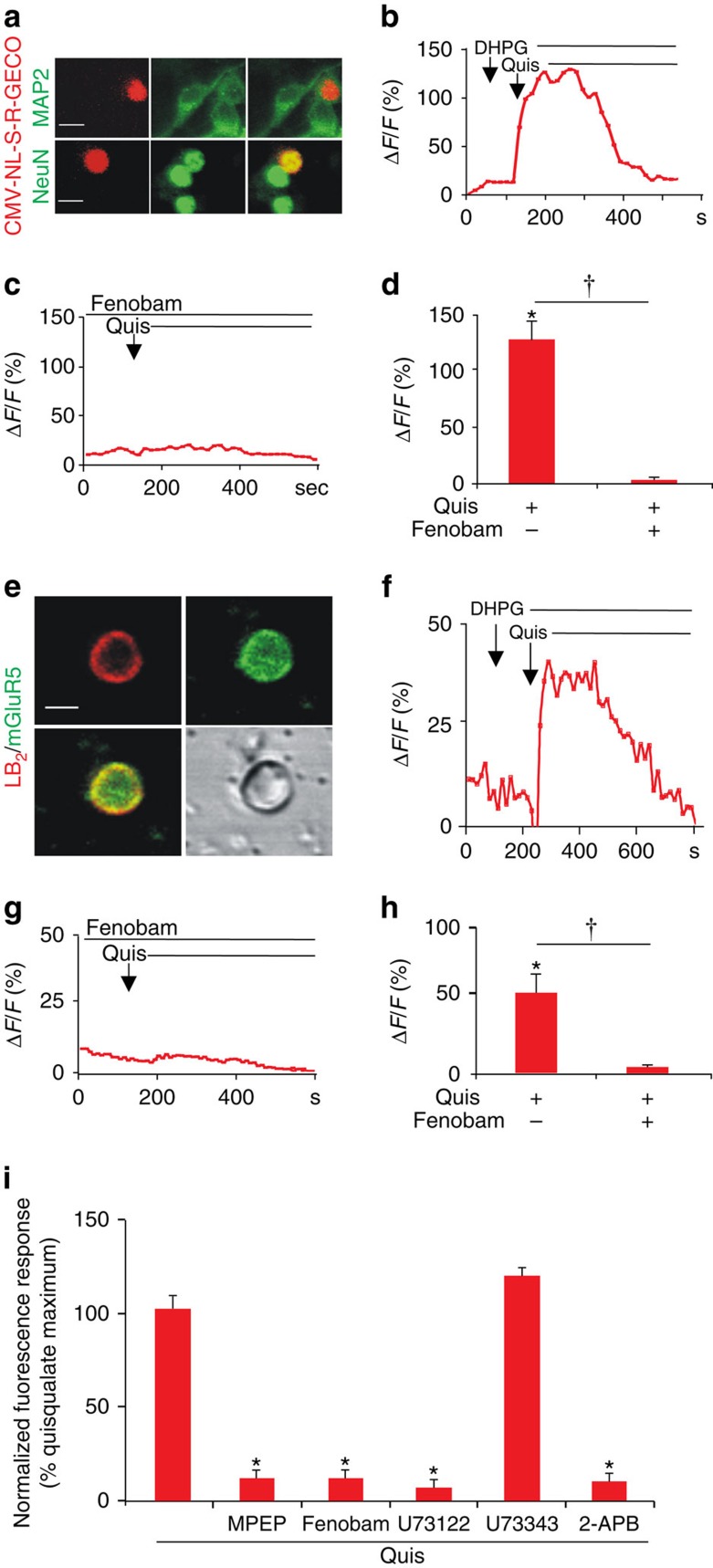
mGluR5-mediated nuclear Ca^2+^ changes in SCDH neurons or isolated nuclei. Cultured rat SCDH neurons were transfected with the nuclear-targeted red fluorescent, genetically encoded Ca^2+^ indicator, CMV-NL-S-R-GECO, on DIV6 and then immunostained or imaged in real time on DIV9. (**a**) Images of SCDH neurons transfected with CMV-NL-S-R-GECO (red) expressed in neuronal cells (as indicated by MAP2 staining; green in the upper panel) and colocalized with the neuronal nuclear marker, NeuN (green in the lower panel) Scale bar, 10 μm. (**b**) Representative trace of nuclear Ca^2+^ responses to agonist stimulation. DHPG application (100 μM) did not induce Ca^2+^ changes whereas quisqualate (Quis, 10 μM) application resulted in sustained nuclear Ca^2+^ rises. (**c**) Fenobam (10 μM) blocked Quis-induced nuclear Ca^2+^ responses. (**d**) Compiled data from peak Δ*F*/*F*_*o*_ (%) with *N*=22 identified nuclei (**b**) and *N*=50 nuclei (**c**) Student's *t*-test, *P<0.05, compared with baseline; ^†^*P*<0.05, Quis alone compared to Quis+fenobam. (**e**) Image of purified nucleus from L4–L6 SCDH stained with lamin B_2_ (*red*) and mGluR5 (green). Scale bar, 5 μm. (**f**) Isolated SCDH nuclei were loaded with Oregon Green BAPTA and imaged to acquire baseline Ca^2+^ changes before agonist application in the presence of AMPA and mGluR1 receptor antagonists. Representative trace of nuclear Ca^2+^ responses to agonist stimulation. DHPG application (100 μM) did not induce Ca^2+^ changes, while quisqualate (Quis, 10 μM) application resulted in sustained nuclear Ca^2+^ rises. (**g**) Fenobam (10 μM) blocked the nuclear Ca^2+^ response induced by Quis. (**h**) Compiled data from peak Δ*F*/*F*_*o*_ (%) with *N*=5 identified nuclei (**f**) and *N*=15 identified nuclei (**g**) Student's *t*-test, *P<0.05, compared with baseline ^†^*P*<0.05, Quis alone compared to Quis+fenobam. (**i**) SCDH cultures (35,000 cells per well) were preincubated with indicated inhibitors (10 μM MPEP, 10 μM Fenobam, 5 μM U73122, 5 μM U73343, 100 μM 2-APB), loaded with Fura-2 AM before EC_80_ quisqualate (5.2 μM) addition and Ca^2+^ flux measurement. Three separate experiments were done in triplicate, Student's *t*-test, *P<0.05, compared to quisqualate alone. All values in figure are expressed as mean±s.e.m.

Since Oregon Green Bapta AM is a global Ca^2+^ indicator dye, the specific contribution of nuclear mGluR5 to intracellular Ca^2+^ changes is equivocal. To address this we performed two experiments. First, SCDH neurons were transiently transfected with a genetically encoded Ca^2+^ indicator (pCMV-NL-S-R-GECO) that is restricted to the cell nucleus (Fig. 2a). Once correct targeting to the nucleus was verified, receptor-mediated Ca^2+^ responses were determined as before. Consistent with the previous data, bath application of the impermeable, non-transported agonist, DHPG, did not induce changes in the nuclear-restricted Ca^2+^ indicator, whereas quisqualate did (Fig. 2b). Pretreatment with the cell permeant mGluR5 antagonist fenobam blocked all quisqualate responses (Fig. 2c,d). To more directly assess nuclear mGluR5 function, isolated nuclei were prepared from P30 L4–6 SCDH. Nuclei were resuspended in intracellular medium, and Oregon Green BAPTA-AM was allowed to accumulate while nuclei were attaching to coated coverslips (Fig. 2e). Although heterogeneous, ∼5 to 10% of isolated nuclei were mGluR5 positive (Fig. 2e). Quisqualate, but not DHPG, induced a sustained Ca^2+^ rise (Fig. 2f) that could be blocked by fenobam (Fig. 2g,h). To confirm the presence of mGluR5 on responding nuclei, coverslips were fixed and processed for mGluR5 immunoreactivity combined with lamin B_2_ staining and then field-relocated immediately following imaging (Fig. 2e). Responsive nuclei were always mGluR5 positive, although not all mGluR5-positive nuclei responded, possibly due to damage in the course of nuclear preparation. Taken together, these data unequivocally establish that nuclear mGluR5 is functionally active.

To test whether activated intracellular SCDH mGluR5 coupled to PLC to generate IP_3_-mediated release of Ca^2+^, we used a fluorescence-based Ca^2+^ imaging plate-reader assay. Cells were preincubated with various inhibitors (10 μM MPEP, 10 μM fenobam, 5 μM U73122 (a PLC inhibitor), 5 μM U73343 (an inactive analog of U73122) or 100 μM 2-APB (an IP_3_R inhibitor) and loaded with Fura-2 AM before Ca^2+^ flux measurement. Results showed that besides being blocked by MPEP and fenobam, mGluR5-mediated Ca^2+^ responses can also be blocked by the PLC inhibitor, U73122 and the IP_3_R inhibitor 2-APB, but not the PLC inactive analogue, U73343 (Fig. 2i). These data show that >90% of SCDH mGluR5 couples to PLC to induce release of Ca^2+^ from intracellular stores associated with IP_3_Rs. Collectively, these data show that intracellular, including nuclear, SCDH mGluR5 can function independently of signals originating at the cell surface and thus plays a dynamic role in mobilizing Ca^2+^ in a specific, localized manner.

### Nerve injury increases nuclear mGluR5 in SCDH neurons

**Figure 3:**
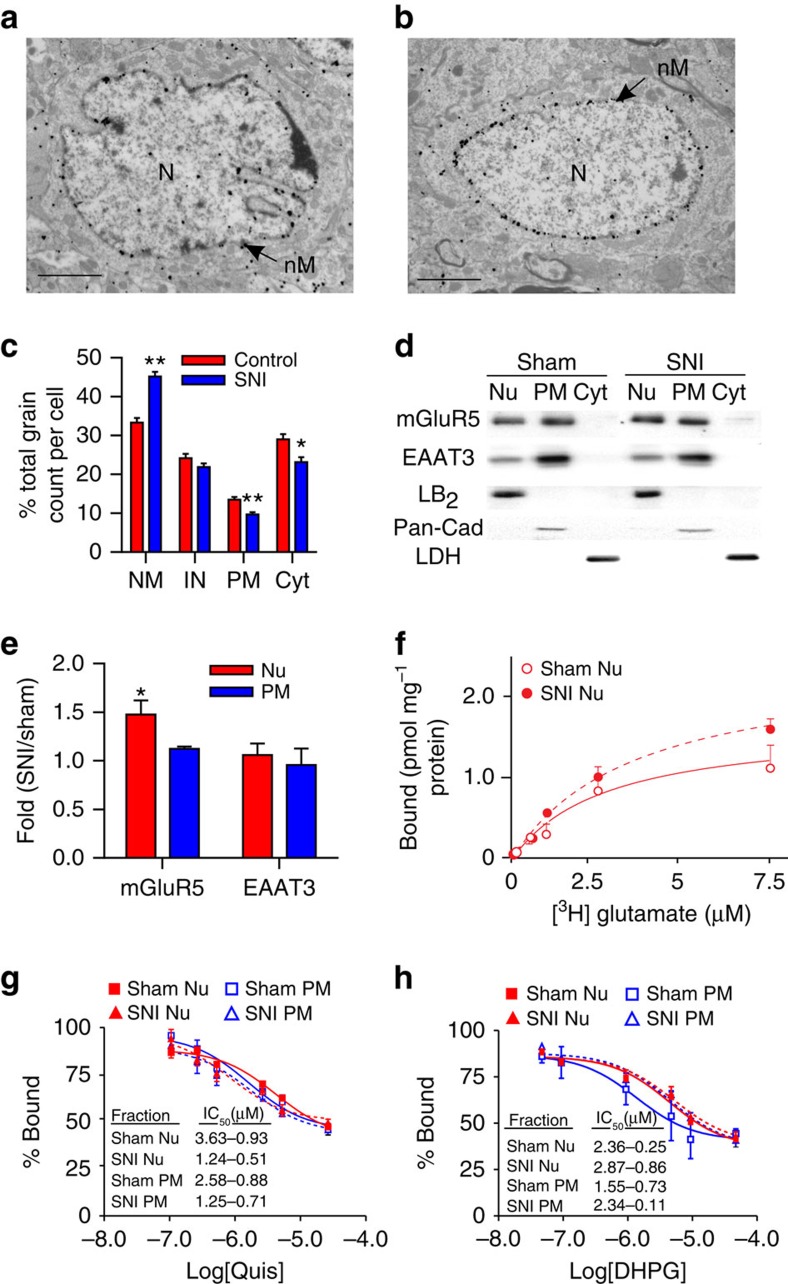
Nerve injury increases SCDH nuclear mGluR5. Electron-micrographs showing increased nuclear mGluR5 in a SCDH neuron of spared-nerve injury (SNI) (**b**) versus sham rat (**a**). (N, nucleus; nM, nuclear membrane). Scale bar, (**a**,**b**) 2 μm. (**c**) Percentage of mGluR5-labelled grains on plasma (PM) or nuclear (NM) membranes, or within cytoplasm (Cyt) or the intranuclear (IN) compartment in SNI and control rats (103 somata were counted from three SNI rats and 86 somata were counted from two control rats, ANOVA *P<0.01; **P<0.001). (**d**) Western blot of nuclear (Nu), cytoplasmic (Cyt) and plasma membrane (PM) fractions from sham and SNI lumbar SCDH. mGluR5 is increased in nuclear (Lamin-B_2_ (LB_2_)) but not PM (Pan-cadherin (Pan-Cad)) or Cyt (lactate dehydrogenase (LDH) fractions from SCDH of SNI rats, quantified in (**e**). (**e**) Data shown represent the mean of three experiments, Student's *t*-test *P*<0.05. (**f**) There are significantly more ^3^H-glutamate sites in SNI nuclear preparations (dashed line) with respect to sham nuclei (solid line) (SNI Bmax=2.87±0.15 pmol mg^−1^, sham Bmax=1.96±0.21 pmol mg^−1^, *P<0.05) (**g**,**h**) percentage binding and IC_50_ values of Quis (**g**) or DHPG (**h**) on nuclear or PM are comparable in sham and SNI animals. Data shown represent the mean of three experiments, comparisons with Student's *t*-test. All values in figure are expressed as mean±s.e.m.

To test whether intracellular mGluR5 contributes to the known role of this receptor in neuropathic pain we used the rodent SNI model which mimics various aspects of human neuropathic pain, inducing spontaneous pain, allodynia and hyperalgesia (Supplementary Fig. 2a–d)^21^. Ultrastructural analysis showed that the percentage of mGluR5 associated with the nuclear membrane increased in SCDH neurons from SNI versus control rats (Fig. 3a–c). Conversely, plasma membrane and intracellular mGluR5 decreased in SNI rats, whereas the percentage of intranuclear mGluR5 remained unchanged (Fig. 3a–c). Western blotting of subcellular fractions from L4–L6 SCDH supported the electron microscopy data showing increased levels of mGluR5 in SNI versus sham nuclear fractions (Fig. 3d,e). In contrast, there was no change in EAAT3 levels across membrane fractions or treatment conditions (Fig. 3d,e). Although reactive astrogliosis is observed after SNI, there was very little colocalization of mGluR5 and the astrocyte marker GFAP in SCDH of either sham or SNI rats (Supplementary Fig. 2e).

Consistent with the above results, increased numbers of mGluR5 binding sites were found in SNI, but not sham SCDH nuclear membranes (Bmax of 2.87±0.15 pmol mg^−1^ versus 1.96±0.21 pmol mg^−1^; Fig. 3f). However, glutamate binding assays showed no significant differences in receptor affinity for DHPG or quisqualate using plasma membrane or nuclear membrane fractions derived from either sham or SNI rat tissues (Fig. 3g,h). These results suggest that nuclear mGluR5 has the same affinity for binding ligands as plasma membrane receptors. One caveat to these assays is that DHPG and quisqualate only displaced ∼50% of glutamate. This is surprising since the conditions used here were identical to protocols used previously to determine half-maximal inhibitory concentrations (IC_50_s) for DHPG and quisqualate in hippocampal and striatal preparations^7^,^8^,^9^,^10^. In those experiments radiolabelled glutamate displacement was between 75 and 80%, and the derived IC_50_ values were essentially the same for DHPG and quisqualate using nuclear and plasma membrane (PM) fractions in striatal and hippocampal preparations^7^,^9^, as shown here for SCDH. Although we have blocked other glutamate binding sites (NMDA, AMPA, kainate and mGluRs), there may be other sites we have not accounted for. Nonetheless, the derived values are similar to those published by ourselves^7^,^9^ and others^23^. Taken together, these results demonstrate increased levels of nuclear mGluR5 in SCDH neurons of neuropathic animals, implicating a pathophysiological role of intracellular mGluR5 in neuropathic pain.

### Nerve injury increases nuclear signalling

**Figure 4:**
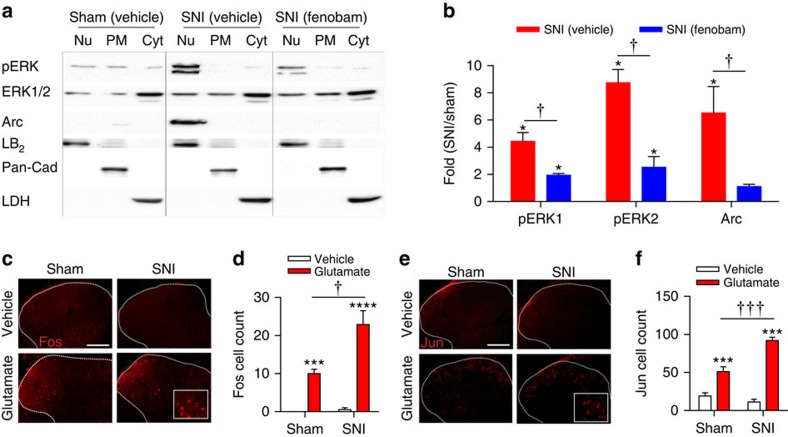
Nerve injury increases SCDH nuclear signalling molecules. (**a**) Western blot analysis of pERK1/2 and Arc/Arg3.1 expression in nuclear (Nu), plasma membrane (PM) and cytosolic (cyt) fractions of SCDH from sham, spared-nerve injury (SNI), and SNI+fenobam rats. (**b**) Quantification reveals increased pERK1, pERK2 and Arc/Arg3.1 in nuclear fractions of SNI rats compared with sham rats (*P<0.05). Fenobam (100 nmol, i.t.) treatment significantly reduced these responses (^†^*P*<0.05). Data shown represent the mean of three experiments, Student's *t*-test. (**c**,**e**) Representative Fos/Jun in ipsilateral SCDH (outlined with dashed lines) of sham and SNI animals following spinal injection of vehicle or glutamate (400 μg), with higher power insets. Scale bar, 100 μm. (**d**,**f**) Fos and Jun are increased in the ipsilateral SCDH of sham rats and further increased in SNI rats (ANOVA ***P<0.001, with respect to vehicle; ^†^*P*<0.05, ^†††^*P*<0.001 with respect to sham). 6–12 sections were averaged for each animal, with *N*=6 animals per group. All values in figure are expressed as mean±s.e.m.

mGluR5 activation leads to phosphorylation of ERK1/2 in SCDH^20^,^24^ and increased activity-regulated cytoskeletal-associated protein (Arc/Arg3.1) in striatum^12^. However, it is unknown if these effectors are activated by intracellular mGluR5 in SCDH. Using subcellular fractionation followed by western blotting of tissue derived from L4–L6 SCDH, we found increases in phosphorylated-ERK1 (pERK1, 4.4-fold increase) and pERK2 (8.7-fold increase) in nuclear fractions from SNI rats as compared with sham rats after normalization with total ERK1/2 (Fig. 4a,b). Both pERK1 and 2 were very low in cytoplasmic fractions, and along with total ERK1/2 showed no difference between groups (Fig. 4a). Arc/Arg3.1 levels in the SCDH nuclear fractions of SNI rats were also increased compared with sham rats (6.5-fold increase; Fig. 4a,b). These results indicate that there is a concomitant increase in mGluR5 (Fig. 3a,b) and enhanced activation of downstream signalling proteins, pERK1, pERK2 and Arc/Arg3.1 in the nuclear compartment in SCDH of SNI rats versus shams (Fig. 4a,b). Importantly, these effects were mGluR5-specific since SNI-induced mGluR5 levels, pERK1, pERK2 and Arc were all significantly reduced by fenobam (Fig. 4a,b).

In addition to triggering pain behaviours^25^,^26^ (Supplementary Fig. 2b,c), spinal administration of glutamate induces a mGluR5-dependent expression of transcription factors such as c-*fos* in SCDH neurons^26^,^27^. However, the relative contributions of intracellular versus plasma membrane mGluR5 to spinal transcription factor expression are unknown. Here we found that Fos and Jun were both increased in the SCDH ipsilateral (Fig. 4c–f) and contralateral (Supplementary Fig. 3a–d) to the nerve surgery 45 min after intrathecal injection of 400 μg glutamate in sham and SNI rats. Importantly, both gene products were significantly higher in the ipsilateral SCDH of SNI versus sham animals (Fig. 4c–f), paralleling increased glutamate-induced pain behaviours in SNI rats (Supplementary Fig. 2c,d). Taken together, increases in both glutamate-induced pain behaviours and transcription factor expression in SNI rats suggest that enhanced responses to spinal glutamate contributes to neuropathic pain. We next ask whether increased levels of intracellular mGluR5 observed in neuropathic animals are responsible for these effects.

### Intracellular mGluR5 blockade reduces pain and c-*fos*

**Figure 5:**
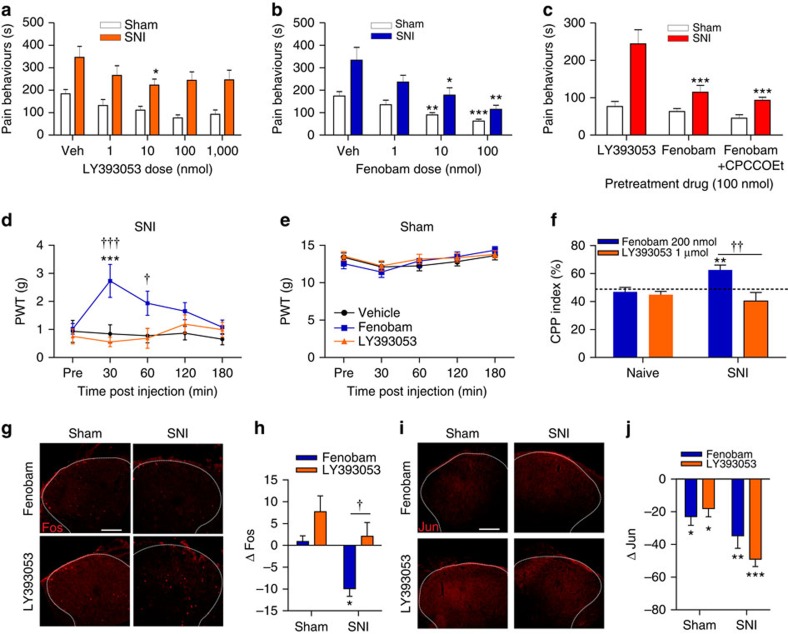
Membrane permeable mGluR5 antagonist reduces pain and Fos. (**a**) LY393053 (1–1,000 nmol) weakly attenuates glutamate-induced pain behaviours in SNI rats (at the 10 nmol dose only, *P*=0.0362), but not sham rats (vehicle, veh). *N*=6 rats per group. (**b**) Fenobam (1–100 nmol) dose-dependently attenuates glutamate-induced pain behaviours in both sham and SNI rats (ANOVA *P<0.05, **P<0.01, ***P<0.001). *N*=6 rats per group. (**c**) Fenobam (100 nmol) or fenobam+CPCCOEt (100 nmol) reduce glutamate-induced pain behaviours significantly more than LY393053 (100 nmol) in SNI rats. (ANOVA ***P<0.001 with respect to LY393053). Conversely, drug effects are equivalent in sham rats. *N*=6 rats/group. Fenobam/LY393053 (100 nmol) data repeated from **a** and **b**. (**d**) Paw withdrawal thresholds (PWTs) of SNI rats are increased by fenobam, but not LY393053 (each 100 nmol), (ANOVA 30-min *P*<0.001; 60-min *P*=0.0156) ***P<0.001 versus vehicle, ^†^*P*<0.05, ^†††^*P*<0.001 with respect to LY393053 (legend as in **e**) *N*=8 rats per group. (**e**) Neither fenobam nor LY393053 affect PWTs of sham rats. *N*=8 rats per group (**f**) SNI, but not naive, rats show conditioned place preference (CPP) to fenobam (200 nmol), (ANOVA CPP index=62.51%, *P*=0.0099; **P<0.01), but not LY393053 (1 μmol). Consequently, fenobam exhibits significantly greater CPP than LY393053 (^††^*P*<0.01 fenobam versus LY393053). *N*=8 rats/group. (**g**) Representative glutamate-induced Fos in ipsilateral SCDH (outlined with dashed lines) of sham and SNI animals after spinal pretreatment with fenobam or LY393053. Scale bar, 100 μm. (**h**) Glutamate-induced Fos in ipsilateral SCDH of SNI rats is reduced by fenobam (ANOVA *P*=0.0024 versus glutamate), but not LY593053 (**P<0.01 with respect to vehicle). Consequently, fenobam-treated rats exhibited significantly lower Fos than LY393053-treated rats (ANOVA *P*=0.0258 versus LY393053, ^†^*P*<0.05 with respect the LY393053). Fos expression was unaffected by either fenobam or LY393053 in sham rats. Six to 12 sections were averaged per animal, with *N*=6 animals/group. (**i**) Representative glutamate-induced Jun in ipsilateral-SCDH (outlined with dashed lines) of sham and SNI animals following pretreatment with fenobam or LY393053 (100 nmol each). Scale bar, 100 μm. (**j**) Jun is reduced by either fenobam or LY393053 in both sham and SNI rats (ANOVA sham *P*=0.0074; SNI *P*=0.0060) or LY393053 (sham *P*=0.0154; SNI *P*<0.0001). (*P<0.05; ***P<0.001). Six to 12 sections were averaged for each animal, with *N*=6 animals per group. All values in figure are expressed as mean±s.e.m.

To investigate the role of intracellular versus cell surface mGluR5 in neuropathic pain, we tested the *in vivo* effects of permeable and impermeable antagonists on pain behaviours induced by 400 μg of spinal glutamate in sham and SNI rats. Spinal pretreatment with the permeable mGluR5 antagonist fenobam (1–100 nmol) produced a highly significant, dose-dependent reduction of glutamate-induced pain behaviours in SNI rats, whereas pretreatment with the impermeable antagonist LY393053 (1–1,000 nmol; Fig. 5a,b) was less effective. As LY393053 antagonizes both mGluR1 and mGluR5, we also tested a 50:50 mixture of CPCCOEt, a permeable mGluR1 antagonist, with fenobam. Contrary to canonical models, fenobam alone (∼66%), or combined with CPCCOEt (∼70%), produced significantly greater analgesia than LY393053 (∼23%) in SNI rats (Fig. 5c).

To mimic physiological conditions (with no exogenous glutamate added), mechanical sensitivity was assessed in both sham and SNI rats by determining paw withdrawal thresholds (PWTs) to plantar hind paw stimulation with von Frey filaments. SNI rats exhibited allodynia (Fig. 5d), as their PWTs were lower than shams (Fig. 5e). PWTs were evaluated in SNI rats following spinal injection of LY393053, fenobam or vehicle. Treatment with either vehicle or LY393053 did not elevate PWTs in SNI rats (Fig. 5d), whereas fenobam significantly elevated PWTs (reflecting relief of allodynia) for one hour post injection (Fig. 5d). As expected, neither antagonist had a significant effect on PWTs in sham animals (Fig. 5e).

Spontaneous pain was also assessed using a conditioned place preference (CPP) paradigm in which a drug or its vehicle were first paired (in counterbalanced order) with opposite sides of a conditioning chamber with differing visual cues. After four daily pairing sessions (two each with drug or vehicle), the time spent in either chamber, or a neutral connecting compartment, was measured for both naive and SNI rats. We first showed that both groups of rats exhibited a preference for a compartment previously paired with morphine (10 mg kg^−1^; Supplementary Fig. 4a), consistent with its well-established analgesic and rewarding effects^28^. Further CPP experiments demonstrated that fenobam produced a place preference effect (CPP Index significantly above 50%) in SNI, but not in naive, rats (Fig. 5f). However, no such place preference was observed in response to spinal treatment with the impermeable mGluR5 antagonist LY393053 (Fig. 5f), establishing that analgesia was produced by fenobam only. Importantly, SNI rats treated with either fenobam or LY393053 showed no baseline place preference (BPP) before drug pairings (Supplementary Fig. 4b). Collectively, multiple pain behaviour experiments show that intracellular mGluR5 is critical for expression of spontaneous pain and mechanical allodynia in neuropathic rats. These results show for the first time that an intracellular GPCR modulates a behavioural phenotype, and that intracellular availability of a given ligand is an important determinant of its therapeutic efficacy.

To test whether blocking cell surface or intracellular mGluR5 would affect downstream signalling pathways associated with pain behaviours, rats were pretreated with either LY393053 or fenobam before measuring spinal glutamate-induced Fos and Jun expression. Consistent with the pain behaviour results, LY393053 did not attenuate spinal glutamate-induced Fos in ipsilateral dorsal horn of SNI rats, whereas pretreatment with fenobam did (Fig. 5g,h). However, neither LY393053 nor fenobam reduced Fos in the ipsilateral dorsal horn of sham rats (Fig. 5g,h), or the contralateral dorsal horn of sham or SNI rats (Supplementary Fig. 4c,d). Both LY393053 and fenobam were equally effective in attenuating glutamate-induced Jun in the ipsilateral (Fig. 5i,j) and contralateral (Supplementary Fig. 4e,f) SCDH of SNI and sham rats. These results suggest that in neuropathic animals c-*fos* is largely dependent on intracellular mGluR5, whereas c*-jun* is not.

### EAAT3 inhibition reduces pain and c-*fos*

**Figure 6:**
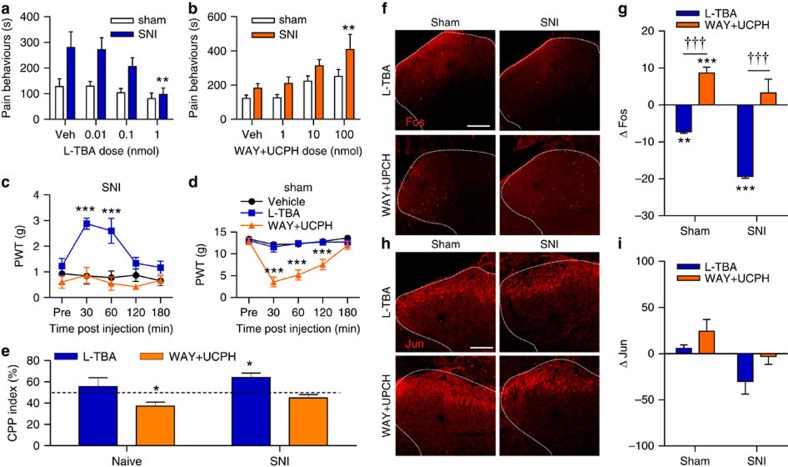
Inhibition of EAAT3 reduces pain and Fos. (**a**) L-TBA (0.01–1 nmol) dose-dependently attenuates glutamate-induced pain in SNI (ANOVA *P*=0.0069), but not sham, rats (vehicle, veh). *N*=6 rats per group. (**b**) WAY+UCPH (1–100 nmol) dose-dependently potentiates glutamate-induced pain in SNI, but not sham, rats (ANOVA *P*=0.003). *N*=6 rats per group. (**c**) Paw-withdrawal thresholds (PWTs) of SNI rats are increased by L-TBA (1 nmol), but not WAY+UCPH (100 nmol) (ANOVA 30 min *P*<0.0001, 60 min *P*=0.0004). (***P<0.001 versus vehicle; legend as in **d**). *N*=8 rats/group. (**d**) PWTs of sham rats are reduced by WAY+UCPH (ANOVA 30–120 min *P*<0.001), but not L-TBA (***P<0.001). N=8 rats per group. (**e**) SNI, but not naive, rats show conditioned place preference (CPP) to L-TBA (1 nmol, CPP index=64.03%, ANOVA *P*=0.0099). Naive rats exhibit conditioned place aversion to WAY+UCPH (ANOVA 100 nmol, CPP index=37.16%, *P*=0.0118 (*P<0.05). *N*=8 rats per group. (**f**) Representative glutamate-induced Fos in ipsilateral SCDH (outlined with dashed lines) of sham and SNI animals following spinal pretreatment with L-TBA (1 nmol) or WAY+UCPH (100 nmol). Scale bar 100 μm. (**g**) For both SNI and sham rats, Fos in the ipsilateral SCDH is reduced by L-TBA (ANOVA *P*=0.0313), but conversely ipsilateral Fos is increased by WAY+UCPH in sham, but not SNI rats (ANOVA *P*=0.0313). (*P<0.05 with respect to vehicle). In both shams and SNI rats, L-TBA attenuates Fos more effectively than WAY+UCPH. (^†††^*P*<0.001 L-TBA versus LY393053). Six to 12 sections were averaged for each animal, with *N*=6 animals per group. (**h**) Representative glutamate-induced Jun in ipsilateral SCDH (outlined with dashed lines) of sham and SNI animals following spinal pretreatment with L-TBA (1 nmol) or WAY+UCPH (100 nmol). Scale bar, 100 μm. (**i**) Neither L-TBA nor WAY+UCPH significantly affected Jun in sham or SNI rats (ANOVA). Six to 12 sections were averaged per animal, with *N*=6 animals per group. All values in figure are expressed as mean±s.e.m.

As intracellular mGluR5 appears essential for the expression of neuropathic pain, we hypothesized that blocking ligand entry into SCDH neurons would also alleviate pain behaviours. In SCDH glutamate is primarily taken up by sodium-dependent transporters including the neuronal EAAT3 (EAAC1; Slc1a1), glial EAAT1 (GLAST, Slc1a3) and glial EAAT2 (GLT-1; Slc1a2)^29^. Previously, spinal administration of the pan-EAAT inhibitor, TBOA, was shown to be pronociceptive in naive animals^30^, but antinociceptive in animals with persistent pain^31^,^32^,^33^. We show here that TBOA had similar paradoxical effects on spinal glutamate-induced pain behaviours in sham versus SNI rats (Supplementary Fig. 5a). Improved EAAT ligand specificity allowed us to selectively test the contributions of either neuronal or glial transporters in sham and neuropathic rats. The EAAT3 specific inhibitor L-ß-threo-benzyl-aspartate (L-TBA, 0.01–1 nmol) was used to block neuronal uptake of glutamate, whereas WAY213613 and UCPH-101 (WAY+UCPH; 1–100 nmol) were used to block EAAT1 and 2, respectively. Pain behaviours induced by 400 μg of spinal glutamate were recorded 10 min following administration of neuronal or glial EAAT inhibitors. After intrathecal L-TBA, a dose-dependent decrease in glutamate-induced pain behaviours was observed in SNI rats, but not sham animals (Fig. 6a). In contrast, intrathecal treatment with a 50:50 mixture of glial EAAT1,2 inhibitors, produced a dose-dependent increase in pain behaviours in SNI rats (Fig. 6b). These results are consistent with the hypothesis that the accessibility of glutamate to intracellular mGluR5 is critical for enhanced pain behaviours to spinal glutamate in SNI rats.

Spinal injection of L-TBA (1 nmol) also attenuated ipsilateral mechanical allodynia for 1 h following injection in SNI animals (Fig. 6c), whereas no change in the PWTs was observed in sham rats (Fig. 6d). Conversely, spinal administration of WAY+UCPH produced no change in the PWTs of SNI rats (Fig. 6c), but induced a very significant reduction in PWTs (or induced allodynia) in sham rats for 2 h (Fig. 6d). CPP experiments demonstrated that 1 nmol of spinal L-TBA produced an analgesic effect (CPP Index significantly above 50%) in SNI, but not in naive, rats (Fig. 6e). In contrast, the EAAT1,2 inhibitors failed to produce a place preference in SNI rats, producing instead a significant place aversion in naive rats (Fig. 6e). Importantly, the rats in either the neuronal or glial EAAT inhibitors groups showed no BPP before drug pairings (Supplementary Fig. 5b). Taken together, these results demonstrate that intracellular transport of glutamate contributes significantly to spontaneous pain and mechanical hypersensitivity in SNI rats, consistent with our hypothesis that increased intracellular mGluR5 plays a role in neuropathic pain.

Spinal glutamate-induced changes in Fos/Jun were also tested after blockade of neuronal and glial transporters. Pretreating rats with L-TBA (1 nmol) 10 min before administering glutamate (400 μg) reduced Fos in the ipsilateral and contralateral SCDH of SNI and sham rats (Fig. 6f,g, Supplementary Fig. 5c,d). Although Jun expression is lower after L-TBA, it was not significantly reduced in any condition (Fig. 6h,i, Supplementary Fig. 5e,f). These results suggest that both spinal glutamate-induced pain and c-*fos* in SNI rats depend on the access of glutamate to intracellular mGluR5.

In contrast, increasing synaptic glutamate by spinal pretreatment with WAY+UCPH resulted in an increase in spinal glutamate-induced Fos in the ipsilateral SCDH of sham, but not SNI rats (Fig. 6f,g), and was not affected in the contralateral SCDH of either sham or SNI rats (Supplementary Fig. 5c,d). Spinal glutamate-induced Jun was not changed by pretreatment with EAAT1,2 inhibitors in either sham or SNI rats ipsi- (Fig. 6h,i) or contralaterally to the nerve surgery (Supplementary Fig. 5e,f). These results indicate that in SNI rats impeding glutamate clearance from the extracellular space by blocking EAAT1,2 induces more pain behaviours and more c-*fos* in response to spinal glutamate injection.

To exclude the possibility that pan-EAAT inhibitors produce antinociceptive effects in neuropathic animals via previously proposed reverse-operation of glutamate transporters^31^, *in vivo* microdialysis was used in conscious behaving animals following inhibition of glial EAATs with WAY+UCPH. As would be expected with normal operation of glutamate transporters, glial EAAT inhibition produced an increase in noxious stimulus-induced glutamate concentration in the SCDH of both sham and SNI rats (Supplementary Fig. 5g).

## Discussion

Some GPCRs, like mGluR5, are localized on intracellular membranes where, *in vitro*, they trigger unique signalling effects^9^,^12^. Here we found that a membrane permeable agonist activating intracellular SCDH mGluR5 produced sustained Ca^2+^ responses, whereas an impermeable, non-transported agonist produced transient Ca^2+^ peaks. Identical responses were observed in SCDH neurons expressing a genetically encoded Ca^2+^ indicator restricted to the nucleus, as well as in acutely isolated SCDH nuclei. When activated, the peak amplitude of nuclear Ca^2+^ responses was ∼40% higher and ninefold greater than at surface mGluR5. Akin to striatal^10^ and hippocampal^7^ receptors, intracellular mGluR5 uses the canonical PLC/IP_3_R signalling pathway to play a dynamic role in mobilizing Ca^2+^ in a specific, localized manner. Using ultrastructural, cellular and pharmacological techniques, we also showed that nerve injury increases nuclear mGluR5 levels, along with the synaptic plasticity effectors pERK1, pERK2, Arc/Arg3.1 and c-*fos*. Behaviourally, blocking only cell-surface mGluR5 with an impermeable antagonist had little effect on neuropathic pain assays, whereas inhibiting intracellular mGluR5 using a permeable antagonist markedly reduced all pain indices and pERK1, pERK2, Arc and c-*fos* expression. Consistent with intracellular mGluR5 driving pain behaviour, blocking glutamate entry into SCDH neurons also produced analgesia and decreased c-*fos*, whereas blocking glial glutamate transporters increased pain behaviours and c-*fos*. To our knowledge, these are the first experiments demonstrating a role for an intracellular GPCR in an *in vivo* behavioural model (see schematic summary diagram in Supplementary Fig. 6).

Although many GPCRs are found on nuclear membranes (for example, receptors for epinephrine, endothelin, platelet-activating-factor and bradykinin), deducing the functional significance of such receptors remains challenging because of limited techniques to probe the nucleus *in situ*, and since most GPCRs are also present at the cell surface. One exception is the α_1A_-adrenoceptor, which is only detected on nuclear membranes in cardiac myocytes^4^, where binding results in PKC activation and translocation leading to troponin phosphorylation and sarcomere shortening^4^. A caged cell-permeable analog of endothelin-1 was used to detect nuclear endothelin receptor-mediated increases of nucleoplasmic Ca^2+^ in cardiac myocytes after intracellular uncaging^5^. Also, activation of the GPCR F2Rl1 anchored at plasma membranes triggered the expression of *Ang1*, whereas nuclear-activated F2Rl1 induced *Vegfa* in retinal ganglion cells^6^. Despite these observations, until now *in vivo* behavioural outcomes resulting from activation of endogenous intracellular receptors have not been assessed.

Given that mGluR5 is an important target, many drugs have been optimized for mGluR5 selectivity, affinity, and pharmacokinetic parameters. Although recent compounds^34^,^35^,^36^ have been developed that overcome the off-target effects^35^ and short-half-lives^36^ of earlier drugs, little emphasis has been placed on which receptor pool ligands act. In blocking cell surface mGluR5, the impermeable antagonist, LY393053, demonstrated only weak analgesia in pain models examined here. In contrast, the cell permeable antagonist, fenobam (a negative allosteric modulator that has both non-competitive antagonist and inverse agonist activity) significantly reduced mechanical allodynia, glutamate-induced pain and c-*fos* expression in neuropathic rats. This suggests that drugs interacting with intracellular mGluR5 are superior against neuropathic pain to those acting at cell surface mGluR5. Also, while fenobam (IC_50_ 80 nM)^37^ is more potent than LY393053 (IC_50_ 1.6 μM)^38^,^39^, we did not see increased analgesic activity when the intrathecal dose of LY393053 was increased from 10 nmoles to 1 μmole suggesting that its analgesic effects plateaued at 10 nmoles. Indeed, the reported IC_50_ values for LY393053 may be unavoidably high, since this impermeable antagonist was assessed using an assay (PI hydrolysis) that employed the transportable mGluR5 agonist quisqualate^38^,^39^. Importantly, these same investigators reported an *in vivo* ED_50_ for LY393053 of 654–955 fmoles against DHPG-induced PI hydrolysis (that is, when the agonist is impermeable) and 9 nmoles against DHPG-induced seizures, when administered centrally as we did here. The fenobam-induced analgesic CPP in neuropathic, but not naive, rats suggests that agents acting at intracellular mGluR5 may produce analgesia with low potential for abuse. In contrast, LY393053's lowering of c-*jun* in SNI rats is consistent with its analgesic effects in acute inflammatory pain^38^,^40^, and suggests a minor contribution from cell-surface mGluR5 in persistent pain. That LY393053 reduced glutamate-induced c-*jun*, but not c-*fos*, while fenobam reduced both, confirms in an *in vivo* model, our previous demonstration *in vitro* that separate intracellular cascades are triggered by cell surface and intracellular mGluR5. Specifically, we previously showed in striatal cultures^7^,^9^,^12^ that cell surface mGluR5 stimulates CAMKIV, p-CREB and c-*jun*, while intracellular mGluR5 phosphorylates ERK1/2 and Elk-1, and enhances c-*fos*, erg-1 and Arc/Arg3.1. The importance of intracellular mGluR5 for c-*fos* induction was further supported here by the significant reduction of glutamate-induced c-*fos* following EAAT3 inhibition.

EAAT3 inhibition not only replicated the analgesic and c-*fos*-reducing effects of fenobam, but also explains the paradoxical antinociceptive effects of pan-EAAT inhibitors on persistent pain^30^,^31^,^32^,^33^. Here a selective EAAT3 inhibitor produced antinociception in SNI rats, whereas EAAT1,2 inhibitors produced pronociception, with similar diverging effects on glutamate-induced pain, mechanical allodynia, CPP and glutamate-induced c-*fos* expression. Our c-*fos* studies, explain recent results showing that intracisternal TBOA significantly increased noxious heat-induced Fos immunoreactivity in the medullary dorsal horn of naive animals, while it significantly reduced this response in animals with earlier inflammation of the vibrissa pad^41^. Thus, TBOA's c-*fos* reducing effects in animals with persistent pain are likely due to EAAT3 blockade, while its c-*fos* enhancing effects in naive animals are likely due to inhibition of EAAT1/2. These results refute an alternative hypothesis proposing abnormal reverse-operation of EAAT1,2 in rats with persistent pain^31^. Indeed, our *in vivo* microdialysis studies demonstrated that EAAT1,2 inhibitors increased spinal extracellular glutamate concentration after noxious stimulation in both sham and SNI rats. These data provide direct evidence that neuropathic pain does not depend on reverse-operation of glutamate transporters; rather analgesia is achieved by blocking the transporters responsible for ligand uptake into SCDH neurons.

Intracellular glutamate concentrations are difficult to assess, although 10 mM is frequently used as a cytoplasmic value with levels ranging up to 100–200 mM within vesicles^42^. However, anti-glutamate immunogold electron microscopy studies indicate that particles representing glutamate are densest in terminal fields reaching 10 mM, whereas far fewer particles are present in somas and/or dendrites and spines (1 mM)^43^,^44^. The latter studies also show large numbers of gold particles over cytoplasmic organelles such as mitochondria, ER and the nucleus^43^,^44^,^45^. These data, combined with glutamate's complex metabolism and the myriad of studies demonstrating that it is highly compartmentalized in neurons^46^,^47^, suggest that there may be far less ‘free' cytoplasmic glutamate than previously suggested. Techniques such as ^13^C-NMR, ^13^C- and/or ^15^N-GC/MS provide compelling evidence that glutamate has many fates within the cell^46^,^47^,^48^. For example, a large proportion of extracellular glutamate is transaminated and enters the mitochondria where it serves as a substrate for the tricarboxylic acid cycle^46^,^47^,^48^. Then too, there is growing precedent for many types of ER–cell surface contacts that might be specialized for given functions^49^,^50^,^51^. Whether such a relationship exists between EAAT3 and mGluR5 is unknown at this moment and awaits further study.

Our electron microscopy studies would suggest that there is more nuclear mGluR5 (∼55%) than there is intracellular (∼25%), thus the sustained Ca^2+^ response should be largely due to a nuclear source. However, we have shown in other studies that hippocampal dendrites^7^ exhibit intracellular mGluR5 responses mirroring the sustained nuclear Ca^2+^ release seen here. Thus, it seems likely that mGluR5 associated with dendritic ER membranes can also proportionately contribute to the intracellular signal. Although it has also been proposed that glial mGluR5 may contribute to glutamate signalling, evidence for mGluR5 in glia comes mostly from studies of cultured glia^52^. Further, although mGluR5 immunostaining has been reported colocalized with markers of astrocytes or microglial in spinal cord, this typically occurs in pathological conditions such as spinal cord injury or amyotrophic lateral sclerosis, particularly when there is reactive gliosis^53^,^54^. Although we see evidence of reactive astrogliosis in SCDH after SNI, we found very little colocalization of staining for mGluR5 and GFAP (astrocyte marker) in either sham or SNI rats (Supplementary Fig. 2e), consistent with an earlier finding showing no change in such colocalization in rat SCDH after nerve root compression^55^.

Although our study emphasizes the physiological significance of the two pools of mGluR5, the mechanism by which nuclear mGluR5 is increased in neuropathic rats remains unknown. Altered trafficking and/or scaffolding are suggested by the significantly decreased plasma membrane and cytosolic mGluR5 and increased nuclear receptors in SNI rats (Fig. 3c). Recent studies suggest the scaffolding proteins Homer 1b/c and Preso1, which interact with mGluR5^56^,^57^,^58^, may be critical. Thus, expression of Homer 1b/c is altered in neuropathic rats^56^, and genetic manipulation of Homer 1b/c or Preso1 significantly affects pain and SCDH Fos^57^,^58^. By further dissecting the effector proteins associated with nuclear mGluR5-dependent processes, more targeted pain therapies can be discovered.

## Methods

### Animals

Adult male Long Evans rats (250–400 g) were used in this study. All experiments were carried out according to ethics protocols approved by McGill University and Washington University Animal Care Committees and followed the guidelines for animal research from the International Association for the Study of Pain (IASP).

### Materials

Glutamate, quisqualate, (*S*)-3,5-dihydroxyphenylglycine (DHPG), L-TBA, TBOA, 6-cyano-7-nitroquinoxaline-2,3-dione (CNQX), D-(−)-2-Amino-5-phosphonopentanoic acid (2*S*)-2-Amino-2-[(1*S*,2*S*)-2-carboxycycloprop-1-yl]-3-(xanth-9-yl) propanoic acid (LY341495), *N*-[4-(2-Bromo-4,5-difluorophenoxy) phenyl]-L-asparagine (WAY 213613), 2-Amino-5,6,7,8-tetrahydro-4-(4-met¬hoxyphenyl)-7-(naphthalen-1-yl)-5-oxo-4H-chromene-3-carbonitrile (UCPH), MPEP, 7-(Hydroxyimino)-cyclopropan [b]chromen-1a-carboxylate ethyl ester (CPCCOEt), and *N*-(3-Chlorophenyl)-*N*′-(4,5-dihydro-1-methyl-4-oxo-1H-imidazol-2-yl)urea (fenobam) were purchased from Tocris Bioscience (Ellisville, MO). THA was obtained from Sigma-Aldrich, (St Louis, MO). 2-Amino-2-(3-*cis*/*trans*-carboxycyclobutyl)-3-(9H-thioxanthen-9-yl) propionic acid (LY393053) was obtained from Lilly Research Laboratories, Eli Lilly and Company (Indianapolis, IN).

### Cell culture and transfection

Primary cultures of SCDH neurons were prepared from postnatal day 1 rat pups as previously described^20^. The cells were plated onto 12-mm poly-D-lysine-coated coverslips for immunostaining or Ca^2+^ imaging or 48-well plates for uptake assays. Cells were cultured in humidified air with 5% CO_2_ at 37 °C for 14–18 days before use. For experiments using the microplate reader, cultures were plated at 35,000 cells per well in black-walled, clear-bottomed 96-well plates and then cultured as above. SCDH cultures were transfected with plasmid pCMV-NLS-R-GECO (Addgene, Cambridge, MA) using Mirus TransIT-X2 (Mirus Bio LLC, Madison, WI) on DIV 6 and then immunostained or imaged in real time on DIV 9.

### Immunocytochemistry of neurons in culture

Spinal neurons were fixed, blocked and incubated with antibodies as described^8^,^9^,^10^. Primary antibodies included polyclonal anti-C-terminal mGluR5 (1:250, Millipore, Billerica, MA, AB5675), anti-NeuN (1:100, Millipore, ABN78), and monoclonal anti-lamin B_2_ (1:100, Invitrogen, Grand Island, NY, 33-2100) and anti-MAP2 (1:500, Millipore, AB5622). Secondary antibodies include goat anti-rabbit (111-165-144) or mouse (115-165-146) Cy3 (1:300, Jackson ImmunoResearch Laboratories, West Grove, PA) and goat anti-rabbit (A-11008) or mouse (A-11029) Alexa-488 (1:300, Invitrogen).

### ^3^H-labelled agonist uptake

[^3^H]-Quisqualate (22.0 Ci mmol^−1^, PerkinElmer Waltham, MA) and l-[^3^H]-glutamate (29.0 Ci mmol^−1^) were used for uptake assay. The SCDH cultures (5 × 10^4^ cells per well) were maintained at 37 °C for 14–18 days before use. Cultured SCDH cells were washed three times in the appropriate buffer (Krebs–Ringer solution containing the following (in mM): 137 NaCl, 5.1 KCl, 0.77 KH_2_PO_4_, 0.71 MgSO_4_·7H_2_O, 1.1 CaCl_2_, 10 D-glucose, and 10 HEPES), Na^+^ free, Cl^−^ free or Na^+^,Cl^−^-free MNDG as described previously^8^) and then incubated at 37 °C in the presence or absence of 100 μM DHPG, 100 μM TBOA, 100 μM THA or 400 μM L-cystine for 15 min before adding labelled agonist. Uptake was terminated after 15 min. Samples were rapidly rinsed three times with ice-cold PBS, solubilized in 150 μl of 1% Triton X-100/PBS, and then analysed by liquid scintillation.

### Fluorescent measurements of intracellular Ca^2+^

Days *in vitro* 14–18 SCDH neurons grown on 12-mm glass coverslips (5 × 10^4^cells per coverslip) were loaded with Ca^2+^ fluorophore, imaged and quantitated as described^8^. SCDH neurons were treated with 100 μM DHPG or 20 μM Quis as well as 10 μM MPEP and/or 20 μM LY393053. Because Quis would also activate AMPA receptors and mGluR1, it was always bath-applied in the presence of 25 μM SYM2206, an AMPA receptor antagonist, and 20 μM CPCCOEt, an mGluR1 antagonist. For consistency SYM2206 and CPCCOEt were also added to controls and DHPG-treated samples. We also used Ca^2+^ flux measurements to assess group responses. In this case primary spinal cord cultures from 1-day-old rat pups were plated at 35,000 cells per well in black-walled, clear-bottomed 96-well plates. After 10–14 days, the cells were loaded with 0.75 μM Fura-2 AM (F14185, Invitrogen) for 30 min at 37 °C and washed with Hanks' balanced salt solution (HBSS). The cells were then preincubated with various inhibitors for 20 min at 37 °C in the assay buffer (HBSS containing 20 μM CPCCOEt and 25 μM SYM2206) before Ca^2+^ flux measurement. Fura-2 fluorescence was measured using a BioTek Synergy H4 Hybrid Microplate Reader. The baseline 340/380 nm excitation ratio for fura-2 was collected for 5 s before injecting 5.2 μM quisqualate. Data were collected for an additional 30 s and then analysed using Biotek's Gen5 analysis software. Percent inhibition of the maximal quisqualate response was calculated by comparing the normalized fold change of the indicator in inhibitor-treated wells to that of controls.

### [^3^H]-glutamate binding assay

L-[^3^H]-glutamate (29.0 Ci mmol^−1^) was obtained from GE Healthcare, Pittsburgh, PA. The fractionated plasma membrane or nuclear pellet was resuspended in buffer containing 40 mM HEPES, pH 7.5, 2.5 mM Ca^2+^, 10 μM CNQX (AMPA/kainate receptor antagonist), 10 μM APV (NMDA receptor antagonist), 20 μM CPCCOEt (mGluR1 antagonist), 100 nM LY341495 (group II & III mGluR antagonist) and protease inhibitors. Incubation was for 60 min at 25 °C, and bound label was separated from free label by fast filtration over #32 filters (Schleicher & Schuell, Keene, NH). Nonspecific binding was determined in the presence of 4 mM glutamate. The binding curves were fit using the GraphPad Prism 3.0 program (Graphpad Software, San Diego, CA, USA).

### Spared-nerve injury

Rats were anaesthetized with isofluorane (2% in 95% O2, 5% CO_2_) and SNI was induced to the left sciatic nerve as previously described^21^. Sham rats received the same surgery except the sciatic nerve was only exposed and received no further manipulation. Sham and SNI rats were tested at 7 days post-surgery, unless otherwise stated.

### Electron microscopy

Pre-embedding immunogold immunocytochemistry was conducted on SCDH sections as described previously^14^. We used a polyclonal anti-mGluR5 antibody (1:400, Millipore, AB5675, lot nos. LV1364844 and LV1416963) directed against a C-terminal sequence of the receptor (specificity and previous use can be found at http://antibodyregistry.org/AB_2295173). Omitting primary antibody was performed as a control (Supplementary Fig. 1c). Preincubation of primary antibody with increasing concentration of synthetic blocking peptide (AG 374, Chemicon) was performed using diaminobenzidine staining and light microscopy (Supplementary Fig. 1d). Subcellular distribution of silver-intensified gold grain labelling, representing mGluR5 antigenic sites, was carried out on electron microscope images of labelled neuronal somata. Grains were counted in four subcellular compartments: plasma membrane, cytoplasm, nuclear membrane and intranuclear. The results were expressed as percentage of mGluR5 in each compartment relative to the total grains in the cell.

### Confocal microscopy

Double labelling of mGluR5 and GFAP was performed on lumbar free floating 50 μm thick transverse sections from SNI and sham rats perfused with 4% paraformaldehyde 2 weeks following the surgical intervention. Briefly, sections were incubated with a mixture of primary antibodies mGluR5 (1/500, Chemicon, AB5675, Lot 2585810) and GFAP (1/2,000, Cell Signaling, 3679, Lot 3) for 48 h at 4 °C. Secondary antibodies consisted of a mixture of donkey anti-rabbit IgG conjugated to Rhodamine Red (1/200, Jackson ImmunoResearch Laboratories, 711-296-152, Lot 103820) and donkey anti-mouse Alexa Fluor 488 (1/500, Molecular Probes, A21202, Lot 49728A). Images were captured with a Zeiss LSM 510 confocal scanning laser microscope using an X63 oil immersion objective and a multitrack scanning method for the detection of both signals.

### Tissue isolation and western blot analysis

Dorsal regions of lumbar spinal cord (L4–L6) were dissected from sham or SNI rats at 1 week or 2 weeks after nerve injury and treatment and resuspended in 20 μl volumes of Buffer ‘A' medium containing 2.0 mM MgCl_2_, 25 mM KCl, 10 mM HEPES (pH 7.5), and protease inhibitors (Complete Tablets; Roche Applied Science, Indianapolis, IN). Tissue was homogenized and nuclei and plasma membranes were prepared as described^11^. Aliquots from each fraction were used for gel electrophoresis as well as membrane binding. Protein concentrations were determined using the Bradford assay (Biorad, Richmond, CA). Fractionated proteins were separated by SDS–PAGE, blotted, and probed with polyclonal anti-mGluR5 (1:1,000, Millipore, AB5675), polyclonal anti-EAAT3 (1:250, Dr J. Rothstein, Johns Hopkins University), monoclonal anti-lamin B_2_ (1:1,000, Invitrogen, Grand Island, NY, 33-2100), polyclonal anti-pan-cadherin (1:1,000, Cell Signaling Technology, Beverly, MA, 4068), polyclonal anti-Lactate Dehydrogenase-Biotin conjugated (1:8,000, Rockland Immunochemicals, Gilbertsville, PA, 200-1673-0100), polyclonal anti-Arc/Arg3.1 (1:500, Synaptic Systems, Germany, 156-003), monoclonal anti-ERK (1:1,000, Cell Signaling Technology, Inc., 4696) and polyclonal anti-pERK (1:2,000, Cell Signaling Technology, Inc., 9101). P-ERK1/2 was normalized to total ERK1/2 and Arc was normalized to Lamin B_2_. A horseradish peroxidase conjugated with goat anti-rabbit IgG (1:2,000, Cell Signaling Technology, Inc., Beverly, MA, 7074) or anti-mouse IgG (1:2,000, Sigma-Aldrich, St Louis, MO, 7076,) was used in conjunction with enhanced chemiluminescence (Clarity Western ECL Substrate, Bio-Rad, Hercules, CA) to detect the signal. Densitometric analyses of proteins were performed using the ChemiDoc MP System together with associated software (Bio-Rad). Full-length western immunoblots are shown in Supplementary Figs 7 and 8.

### Drug administration *in vivo*

All drugs were administered by intrathecal (i.t.) injection at the L2-L5 spinal cord level, while the rat was under isofluorane anaesthesia. Drugs were dissolved either in distilled water (glutamate or morphine) or 5% dimethyl sulfoxide+0.1 M cyclodextrin or 25% dimethyl sulfoxide in distilled water (all other drugs). Except for morphine, which was given subcutaneously (s.c.) in a volume of 1 mg ml^−1^, all drugs were prepared to make an injectable volume of 20 μl for spinal injection. The prepared drugs were sonicated for 45 min to generate a clear solution or a microsuspension. All drugs were prepared fresh on the day of treatment. The drug dosages for later experiments were based on the dose–response curves derived from the glutamate-induced pain behaviour experiments. Unless otherwise stated in the Results section or below, pretreatment drugs were all given 1-week post SNI or sham surgery.

### Nociceptive testing

*Glutamate-induced pain behaviours*. One week post-SNI or sham surgery rats were habituated to an observation chamber (30 × 30 × 30 cm) fitted with a transparent floor under which was placed a mirror to allow an unobstructed view of the animal's paws for 30 min. Following habituation, rats were given two i.t. injections 10 min apart: either a pretreatment drug or vehicle followed by an injection of 400 μg of glutamate. The rats were then returned to the chamber and allowed to move freely. Pain behaviours were measured as the time spent licking the hind paws, lower legs and tail over a 30-min period. The behaviours were recorded starting from when the rats awoke from anesthesia and made their first coordinated movements.

*Mechanical allodynia testing*. Rats were tested for mechanical allodynia between 7 and 17 days after sham or SNI surgery, following each of the five i.t. drug treatments (L-TBA, WAY+UCPH, fenobam, LY393053) or vehicle. Only one drug was tested per day with 1 day in between drug testing, with drug order counterbalanced with a Latin square design. Before each testing session, each rat was habituated to a testing box (17 × 15 × 12 cm) with a wire-mesh grid floor, for a 1-h period. Before drug administration, a baseline was established using von Frey hairs applied through the grid floor to the ventral surface of the hind paw. Each hair was applied for a 10-s period or until the animal withdrew the hind paw without ambulating. During each testing trial, the series of hairs were presented following a validated up–down procedure^59^, and the 50% PWT was calculated for each rat. After a baseline score was established, rats were given either the drug or vehicle via i.t. injection and returned to the testing box. PWTs were measured 30, 60, 120 and 180 min following the injection.

### Conditioned place preference (CPP)

CPP procedures began on day 7 following the sham or SNI surgery. The CPP chamber consisted of two pairing chambers (22 × 38 cm) connected by a third, neutral compartment (44 × 22 cm). On the habituation and test sessions (day 7 and 12 post surgery) the neutral compartment had openings that allowed the rats to freely explore all three chambers. The pairing chambers contained salient visual cues (horizontal versus vertical black and white lines) on the chamber walls and floor. During the habituation session in which the rats were allowed to explore all chambers for 30 min, an initial measurement of BPP was taken by measuring the time spent in each chamber over 15 min. Rats spending >75% of the time in any one chamber at habituation were removed from the experiment. The next day, using a randomized block design, rats were assigned a chamber-drug pairing. Either the drug or the vehicle was administered i.t. (except morphine which was given s.c.) and the rats were restricted to one of the two pairing chambers for a period of 60 min. The following day the rats would receive the other drug in the opposite chamber. On the CPP test day, the rats placed in the CPP chamber with the open gate configuration for 15 min and the time spent in the drug- and vehicle-paired or neutral chamber was again measured. The CPP index was defined as the time spent in the drug-paired chamber divided by the time spent in both the drug- and vehicle-paired chambers multiplied by 100; a CPP >50% indicates a preference for the drug-paired chamber while a CPP <50% indicates an aversion to the drug-paired chamber.

### Immunohistochemistry preparation for Fos and Jun

In Fos and Jun experiments, animals were perfused 45–60 min following glutamate injection. Twenty-micrometre thick cross sections of the lumbar spinal cord were cut using a cryostat (Leica, Wetzlar, Germany) and collected on poly-L-lysine coated superfrost plus slides (Fisher Scientific, PA, USA). The tissue sections were incubated for 1 h at room temperature in 10% normal donkey serum in 0.1% Triton-X in PBS (PBS-Tx) to block unspecific labelling. For Jun labelling, sections were incubated at 4 °C for 48 h using rabbit polyclonal to Jun (1:1,000, Abcam, ab31419). After three rinses in PBS-Tx, the sections were incubated for 72 h at room temperature with donkey polyclonal anti-rabbit IgG conjugated to Rhodamine Red (1:500, Jackson ImmunoResearch Laboratories, 711-296-152, Lot 90396) for Jun, visualization. Separate slides were labelled for Fos using rabbit polyclonal to c-fos (1:5,000, Millipore ABE 457) overnight at 4 °C. After three rinses in PBS, the sections were incubated for 4 h at room temperature with donkey polyclonal anti-rabbit IgG conjugated to Rhodamine Red (1:800, Jackson ImmunoResearch Laboratories, 711-296-152, Lot 90396). Finally, the sections were washed two times in PBS-TX then once in PBS for 10 min and cover slipped with an anti-fading mounting medium (Aqua PolyMount, Polysciences Inc., Warrington, PA). Antibodies were always diluted in PBS-Tx.

### Fos and Jun cell counting

The numbers of Jun-labelled cells were estimated using ImageJ (NIH freeware, Bethesda, MD). Images were first converted into 8-bit format. The threshold command was used to segment the image into labelled cells and background, with particles <100 or >1000 pixels excluded. Fos-labelled cells were counted manually. The average number of Fos- and Jun-labelled cells per section was used as a single data point. All pretreatment+glutamate conditions were compared with vehicle+glutamate by subtracting the mean number of Fos- and Jun-labelled cells in vehicle+glutamate rats from each rat in the pretreatment+glutamate condition to yield a ΔFos and ΔJun cell count for each of the pretreatment drugs.

### Microdialysis and intrathecal catheter

Microdialysis fibres and an intrathecal injection catheter were implanted into rats as described previously^60^. After recovery from surgery, rats were placed in a Raturn Interactive System (Bioanalytic Systems, Inc, West Lafayette, IN, USA) with a tethering system, which allowed tubing from the microdialysis catheter to be connected to a syringe pump on one end and to a refrigerated fraction collector on the other. Sample collection was done as described^60^ with collections taken every 5 min during a 30 min baseline period (with only the final three measurements assayed), for 30 min after spinal injection of either vehicle or a 50:50 mixture of WAY+UCPH (100 nmoles), and for 30 min following intraplantar injection of 1.0% formalin.

### Sampling and statistical analyses

For *in vivo* studies, sample sizes were 6–9 per group, which is commonly required to obtain statistical significance, while *in vitro* studies utilized a minimum of three experimental replicates. In all experiments, animals were randomly assigned to groups, with a randomized block design used for the CPP experiments and randomization performed after BPP measurement. In all experiments, measurements were performed with the experimenter blind to the experimental conditions. All values are expressed as mean±s.e.m., and no samples/animals were excluded from analysis. All statistical tests were performed using GraphPad Prism version 5 for Windows (GraphPad Software, La Jolla, CA or Statistica 6 (Stat Soft, Tulsa, OK) and all reported statistical analyses were justified based on sample size, homogeneity of variance and normal distribution of the data. The behavioural dose–response curves were analysed using between measures two-way analysis of variances (ANOVAs; pain condition × dose) and *post hoc* comparisons between drug dose and vehicle condition, unless stated otherwise, were performed using two-sided Dunnett's test. Paw withdrawal thresholds were similarly analysed by repeated measures ANOVA (drug × time) followed by *post hoc* comparisons between drug conditions at each time point using the Bonferroni test. For electron microscopy quantification, the grain count in each subcellular compartment was expressed as a percentage of the total grain count for that cell. Percentage counts were grouped by animal and condition (control or SNI). A two-way nested model ANOVA was followed by Scheffé *post hoc* comparisons. Immunohistochemical data were analysed using two-way ANOVAs to identify significant changes in cell counts compared with the vehicle pretreatment condition, which was normalized to zero. CPP scores were analysed using two-way ANOVAs with Bonferroni correction for multiple comparisons used to compare CPP index to a value of 50%. All other experiments were analysed using two-way ANOVAs with Bonferroni correction and two-sided tests for multiple comparisons or two-tailed Student's *t*-test for comparing two groups.

## Additional information

**How to cite this article:** Vincent, K. *et al*. Intracellular mGluR5 plays a critical role in neuropathic pain. *Nat. Commun.* 7:10604 doi: 10.1038/ncomms10604 (2016).

## Supplementary Material

Supplementary Information Supplementary Figures 1-8
