# Supplementary material for: Intracellular mGluR5 plays a critical role in neuropathic pain
Source: Nat Commun. 2016 Feb 3;7:10604. doi: 10.1038/ncomms10604 (PMC4742982; doi:10.1038/ncomms10604)
Supplement: Supplementary Information — Supplementary Figures 1-8 [file ncomms10604-s1.pdf]

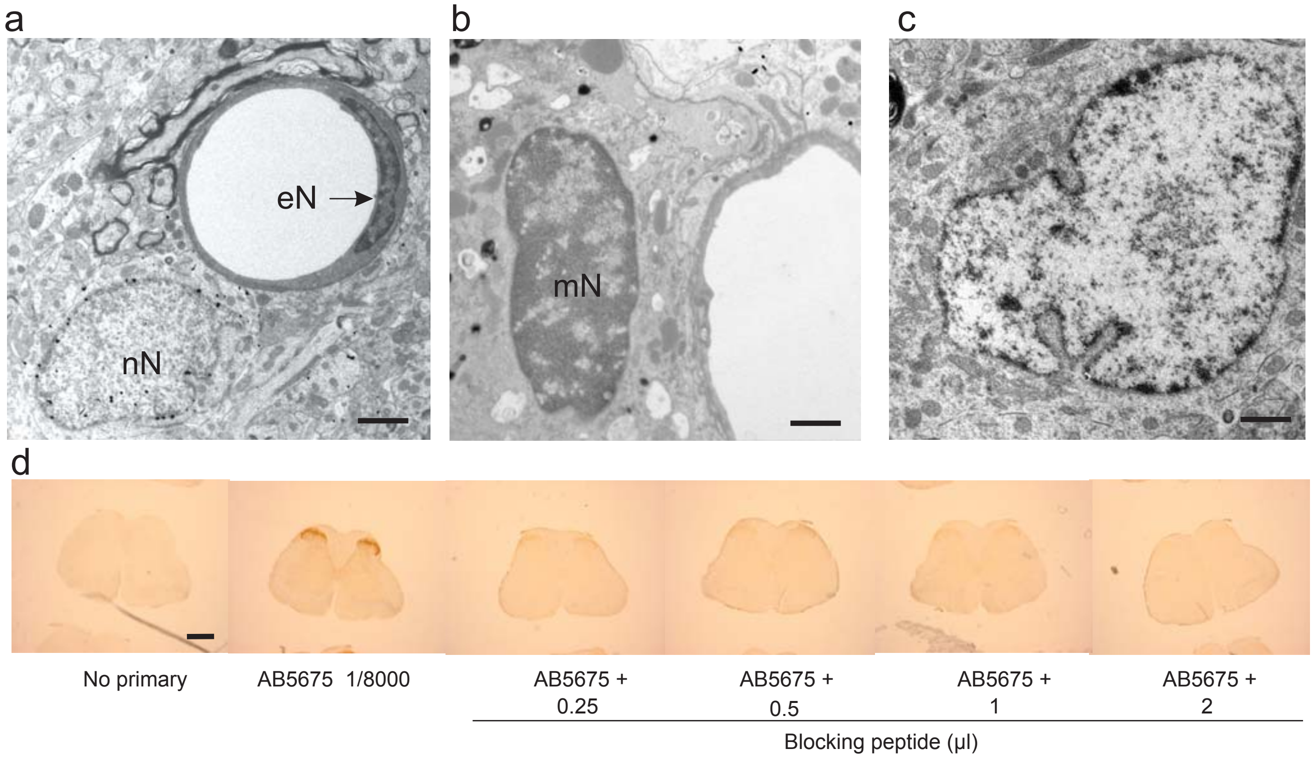

Supplementary Figure 1: EM Images of non-neuronal SCDH cells lacking mGluR5 and tests of specificity of mGluR5 antibody. (**a-b**) EM images showing mGluR5 immunoreactivity (as silver-intensified gold particles) in sections from SCDH. Scale bar 2 µm for **a**-**c**. (**a**) Gold particles are observed in a neuronal nucleus (nN), but not the nucleus of an endothelial cell (eN, endothelial cell nucleus). (**b**) Gold particles are not observed in a microglial nucleus (mN). (**c**) EM image showing no mGluR5 immunogold labelling in a neuronal nucleus in the absence of primary antibody. (**d**) mGluR5 diaminobenzidine staining in lumbar SCDH is decreased and ultimately abolished by preincubation of primary antibody with increasing concentration of specific blocking peptide Ag374. Scale bar 500 µm.

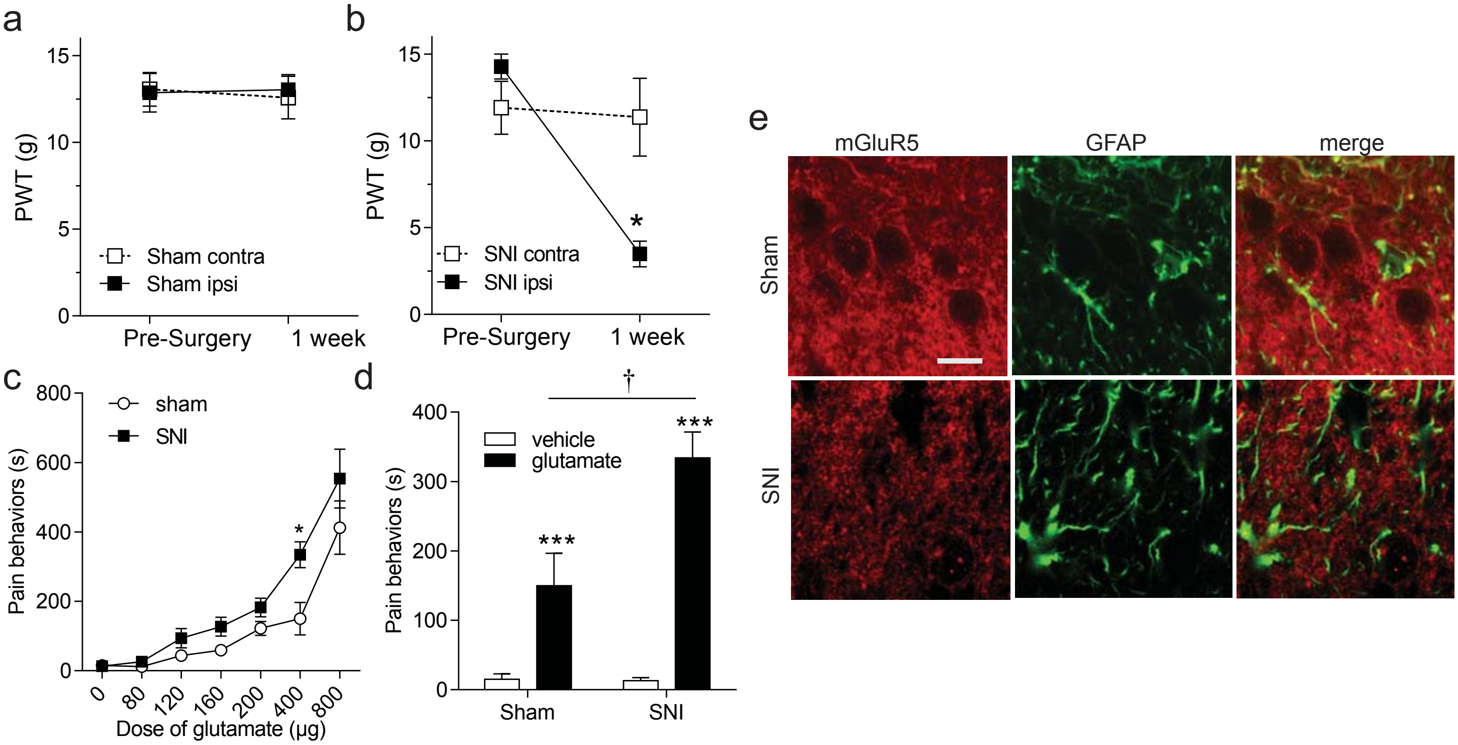

Supplementary Figure 2: Mechanical sensitivity and spinal glutamate-induced pain behaviors, and photomicrographs of mGluR5/GFAP in astrocytes, in sham and SNI rats. (**a**,**b**) Paw-withdrawal thresholds (PWTs) of sham (**a**) and SNI (**b**) rats before and one week after nerve injury. Ipsilateral, but not contralateral, PWTs are significantly reduced one week after SNI (ANOVA , $p=0.0049$), but not sham surgery). N=5 rats/group. (**c**) Dose-dependent pain behaviors induced by spinal glutamate (80-800 µg) in both sham and SNI rats. Glutamate-induced pain behaviors are higher in SNI with respect to sham rats (ANOVA, $p<0.0001$) (* $p<0.05$). N=6 rats/group. (**d**) Histogram showing pain behaviors of sham and SNI rats over a period of 30 min after spinal administration of glutamate (400 µg, black bar) or vehicle (white bar). Pain behaviors are significantly increased by glutamate in sham rats, and to a significantly greater extent in SNI rats (ANOVA, $p=0.0010$). (*** $p<0.001$ with respect to vehicle; † $p<0.05$ with respect to sham). N=6 rats/group. (**e**) Immunoreactive labelling of mGluR5 (left panels, red), the astrocyte marker GFAP (centre panel, green), and double-labelling of mGluR5/GFAP (right panel) in both sham (upper panels) and SNI (lower panels) rats. Scale bar 10 µm. Note that although there is evidence of reactive astrogliosis in SNI rats, there is very little colocalization of mGluR5 and GFAP in either sham or SNI rats.

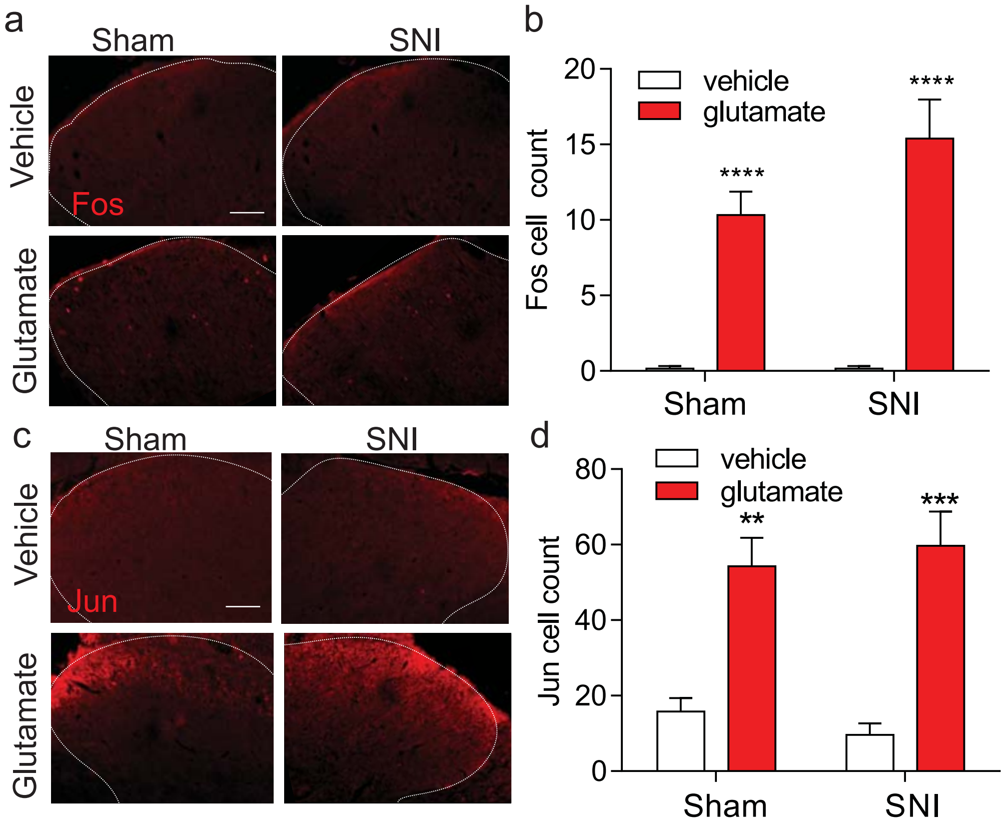

Supplementary Figure 3: Glutamate-induced gene expression in contralateral spinal cord dorsal horn of sham and SNI rats. (**a**) Representative Fos in contralateral SCDH (outlined with dashed lines) of sham and SNI animals following injection of vehicle or glutamate (400 µg). Scale bar 100 µm. (**b**) Glutamate significantly increases Fos in the contralateral dorsal horn in sham and SNI rats (ANOVA, $p<0.0001$). (***$p<0.001$). 6-12 sections were averaged per animal, with N=6 animals/group. (**c**) Representative Jun in contralateral SCDH of sham and SNI animals following injection of vehicle or glutamate (400 µg). Scale bar 100 µm. (**d**) Glutamate significantly increases Jun in the contralateral dorsal horn in sham and SNI rats (ANOVA, $p<0.0001$). (**$p<0.01$; ***$p<0.001$). 6-12 sections were averaged per animal, with N=6 animals/group.

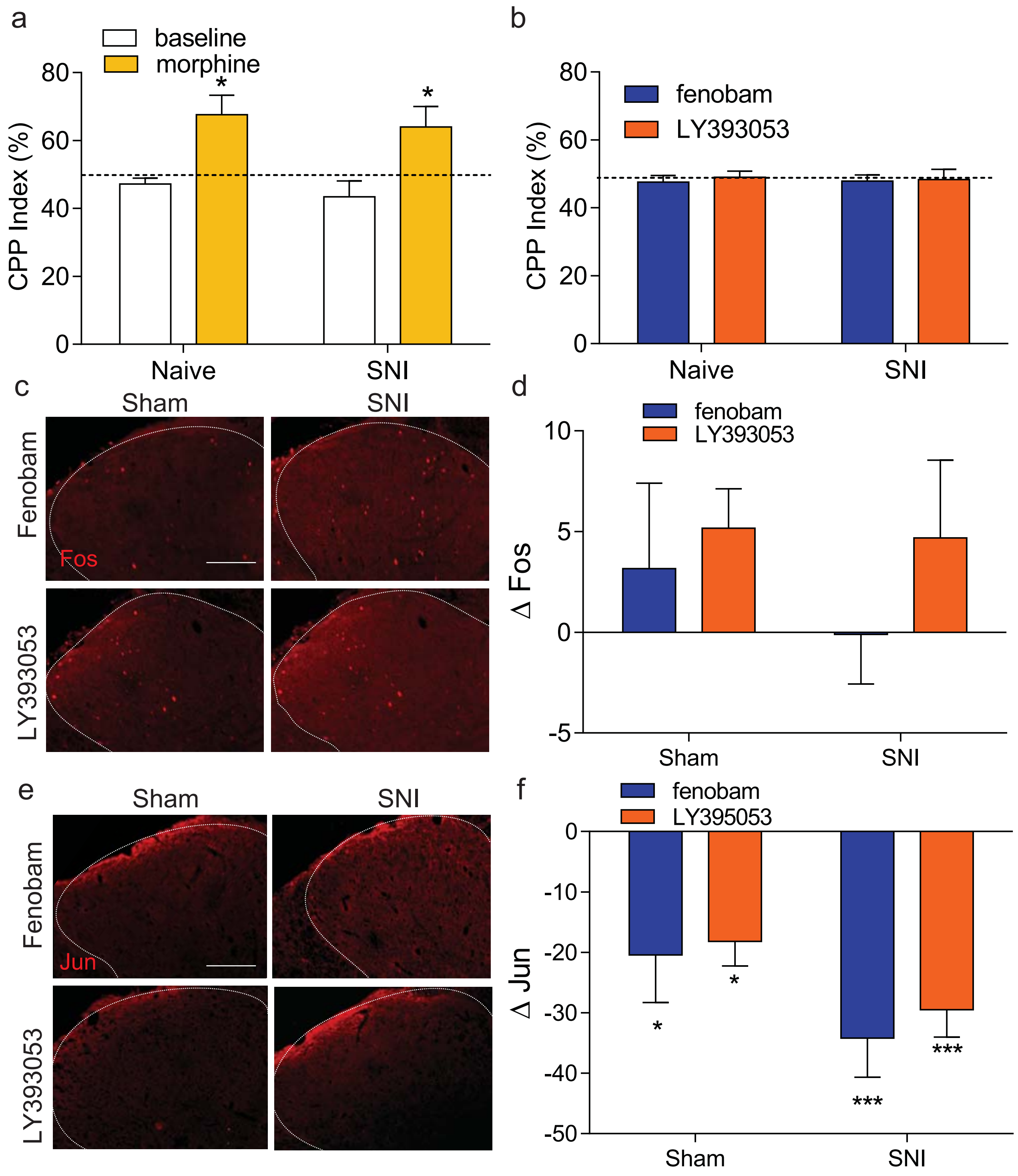

a
baseline
morphine
CPP Index (%)
80
60
40
20
0
*
*
Naive
SNI
b
fenobam
LY393053
CPP Index (%)
Naive
SNI
c
Sham
SNI
Fenobam
LY393053
Fos
d
fenobam
LY393053
Δ Fos
10
5
0
-5
Sham
SNI
e
Sham
SNI
Fenobam
LY393053
Jun
f
fenobam
LY395053
Δ Jun
0
-10
-20
-30
-40
-50
*
*
***
***
Sham
SNI

Supplementary Figure 4: Effects of fenobam and LY393053 on pain behaviors, basal PP and/or gene expression in the contralateral SCDH. (**a**) Morphine (10 mg/kg) produced a conditioned place preference (CPP) following two pairings in both naïve and SNI rats (naive CPP index=67.65%, ANOVA, p=0.0144; SNI CPP index=63.98%, p=0.0489 (BPP, basal PP). N=9 rats/group. (**b**) Before treatment with fenobam or LY393053 (i.e., BPP), neither sham nor SNI rats differed in the time they spent in the two chambers of the apparatus. ANOVA. N=8 rats/group. (**c**) Representative glutamate-induced Fos in contralateral SCDH (outlined with dashed lines) of sham and SNI animals after pretreatment with fenobam or LY393053 (100 nmol each). Scale bar 100 µm. (**d**) Neither fenobam nor LY393053 significantly affected Fos expression in the contralateral SCDH of sham or SNI rats. ANOVA. 6-12 sections were averaged per animal, with N=6 animals/group. (**e**) Representative glutamate-induced Jun in contralateral SCDH (outlined with dashed lines) of sham and SNI animals following pretreatment with fenobam or LY393053 (100 nmol each). Scale bar 100 µm. (**f**) Jun was reduced by either fenobam or LY393053 in both SNI (fenobam p=0.0032; LY393053 p=0.0013, ANOVA) and sham (fenobam p=0.0493; LY393053 p=0.0066, ANOVA) rats. (*p<0.05; ***p<0.001). 6-12 sections were averaged per animal, with N=6 animals/group.

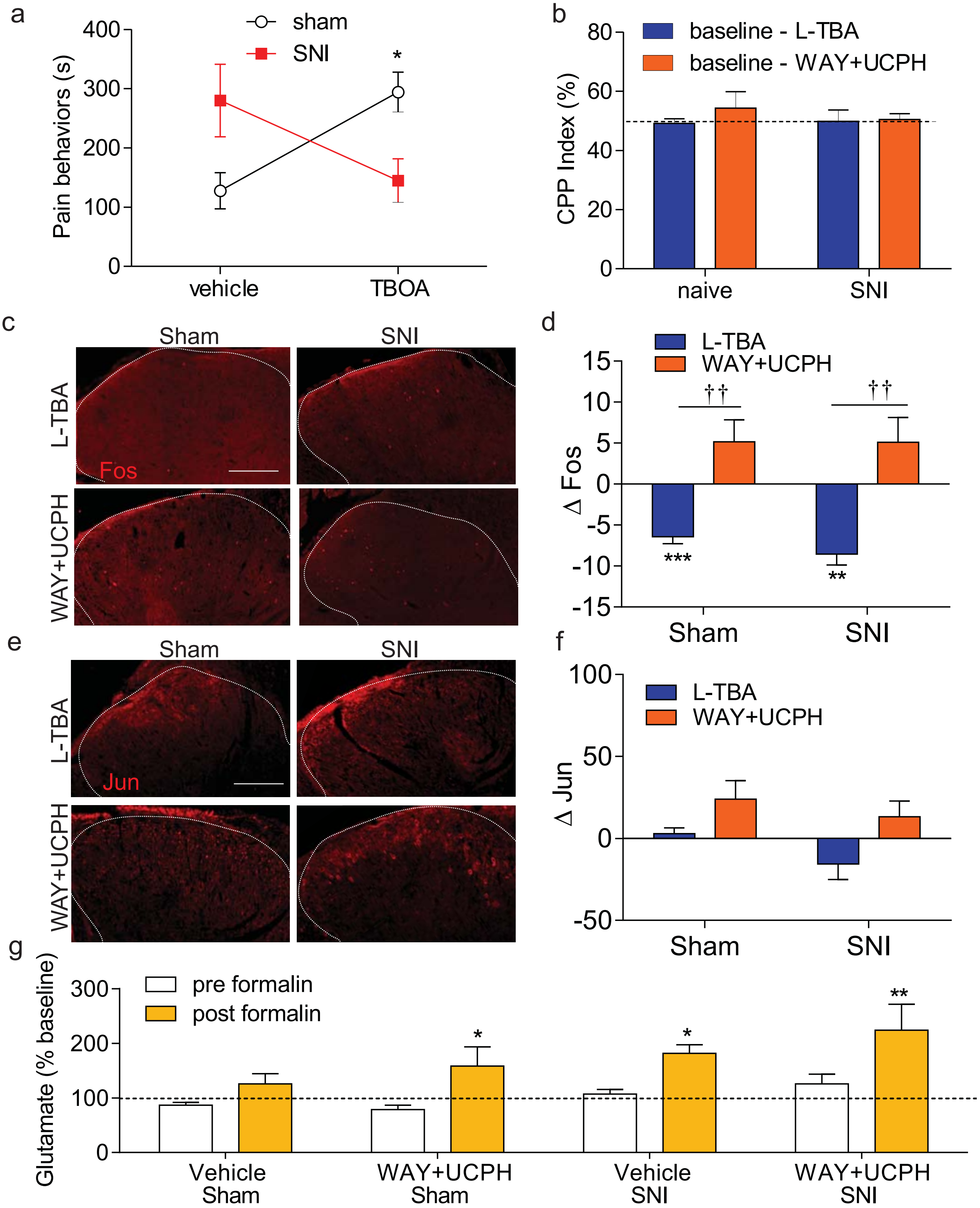

a
sham
SNI
Pain behaviors (s)
400
300
200
100
0
*
vehicle
TBOA
b
baseline - L-TBA
baseline - WAY+UCPH
CPP Index (%)
80
60
40
20
0
naive
SNI
c
Sham
SNI
L-TBA
WAY+UCPH
Fos
d
L-TBA
WAY+UCPH
Δ Fos
15
10
5
0
-5
-10
-15
††
††
***
**
Sham
SNI
e
Sham
SNI
L-TBA
WAY+UCPH
Jun
f
L-TBA
WAY+UCPH
Δ Jun
100
50
0
-50
Sham
SNI
g
pre formalin
post formalin
Glutamate (% baseline)
300
200
100
0
*
*
**
Vehicle Sham
WAY+UCPH Sham
Vehicle SNI
WAY+UCPH SNI

Supplementary Figure 5: Effects of L-TBA or WAY+UCPH on pain behaviors, basal CPP, and/or gene expression in the contralateral SCDH. (**a**) Spinal TBOA (10 nmol) has a significant interactive effect on glutamate-induced pain behaviors in sham and SNI rats (ANOVA, $p=0.0019$). Thus, TBOA significantly potentiates pain behaviors in sham animals ($p=0.0193$) and significantly reduces pain behaviors in SNI rats (ANOVA, $p=0.0317$). *$p<0.05$ with respect to glutamate alone. N=6 rats/group. (**b**) Before treatment with L-TBA or WAY+UPCH, neither naive nor SNI rats differed in the time they spent in the two chambers of the apparatus (BPP, basal place preference). (ANOVA). N=9 rats/group. (**c**) Representative glutamate-induced Fos in the contralateral SCDH (outlined with dashed lines) following pretreatment with either L-TBA or WAY+UCPH (100 nmol each). Scale bar 100 μm. (**d**) Fos in contralateral SCDH was decreased in both sham and SNI rats by pretreatment L-TBA (ANOVA, Sham $p=0.0006$; SNI $p=0.0014$), but was not significantly affected in either sham or SNI rats by pretreatment with WAY+UCPH. (**$p<0.01$ versus vehicle). Consequently, Fos was significantly lower in rats treated with L-TBA as compared to those treated with WAY+UCPH (††$p<0.01$ L-TBA versus WAY+UCPH). 6-12 sections were averaged per animal, with N=6 animals/group. (**e**) Representative glutamate-induced Jun in contralateral SCDH (outlined with dashed lines) of sham and SNI animals following pretreatment with either L-TBA or WAY+UCPH (100 nmol each). Scale bar 100 μm. (**f**) Jun in contralateral SCDH was not significantly affected by either L-TBA or WAY+UCPH in either sham or SNI rats (ANOVA). 6-12 sections were averaged per animal, with N=6 animals/group. (**g**) Changes in glutamate levels following spinal treatment with vehicle or WAY+UCPH (100 nmol) and hind paw formalin injection in sham and SNI rats. In sham rats (N=6), formalin injection increased spinal glutamate concentrations after WAY+UCPH ($p<0.05$), but not vehicle. In SNI rats (N=7), formalin injection increased spinal glutamate both after vehicle (*$p<0.05$) or WAY+UCPH (**$p<0.01$). ANOVA.

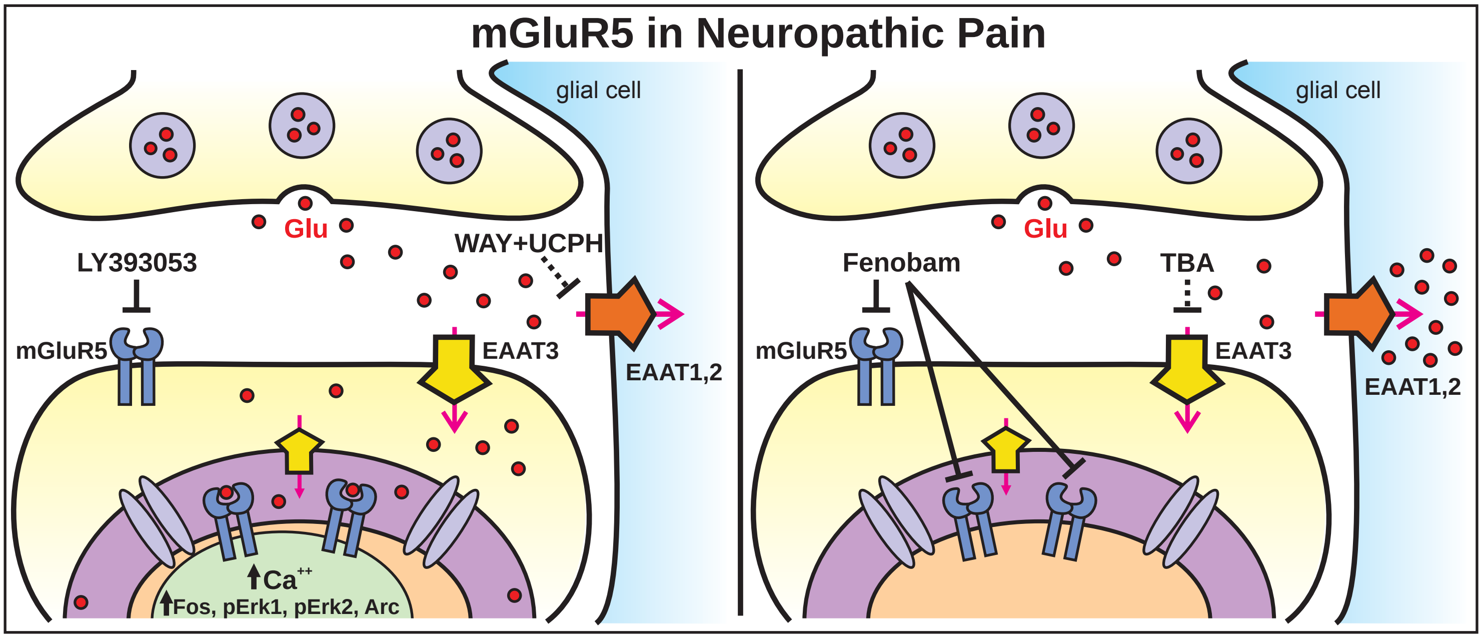

Supplementary Figure 6: Schematic summary illustrating the of targeted mGluR5 treatments on neuropathic pain. Glutamate (Glu) is released from presynaptic terminals and binds at two pools of functional mGluR5), at the cell surface and at intracellular membranes of postsynaptic neurons in spinal cord dorsal horn (SCDH). mGluR5 at the nuclear membrane is upregulated in neuropathic pain conditions and is critical for enhanced nuclear Ca^{2+}-dependent Fos, pERK1, pERK2 and Arc in SCDH. **Left panel**, Selectively blocking cell surface mGluR5 with LY393053 has little effect on neuropathic pain behaviors or Fos. Selectively blocking glutamate uptake into glial cells with excitatory amino acid transporter (EAAT) 1,2 inhibitors (WAY + UCPH) also does not reduce neuropathic pain or Ca^{2+}-dependent Fos, and can even induce pronociceptive effects in neuropathic and/or non-neuropathic conditions (not depicted) due increased synaptic and extrasynaptic glutamate. **Right panel**, Blocking both intracellular and cell surface mGluR5 with fenobam dramatically reduces both neuropathic pain and Ca^{2+}-dependent Fos, ERK1, ERK2 and Arc. Selectively blocking glutamate uptake into neurons with an EAAT-3 inhibitor (TBA) also dramatically reduces neuropathic pain and Fos. Together these results highlight the importance of intracellular mGluR5 in SCDH to neuropathic pain.

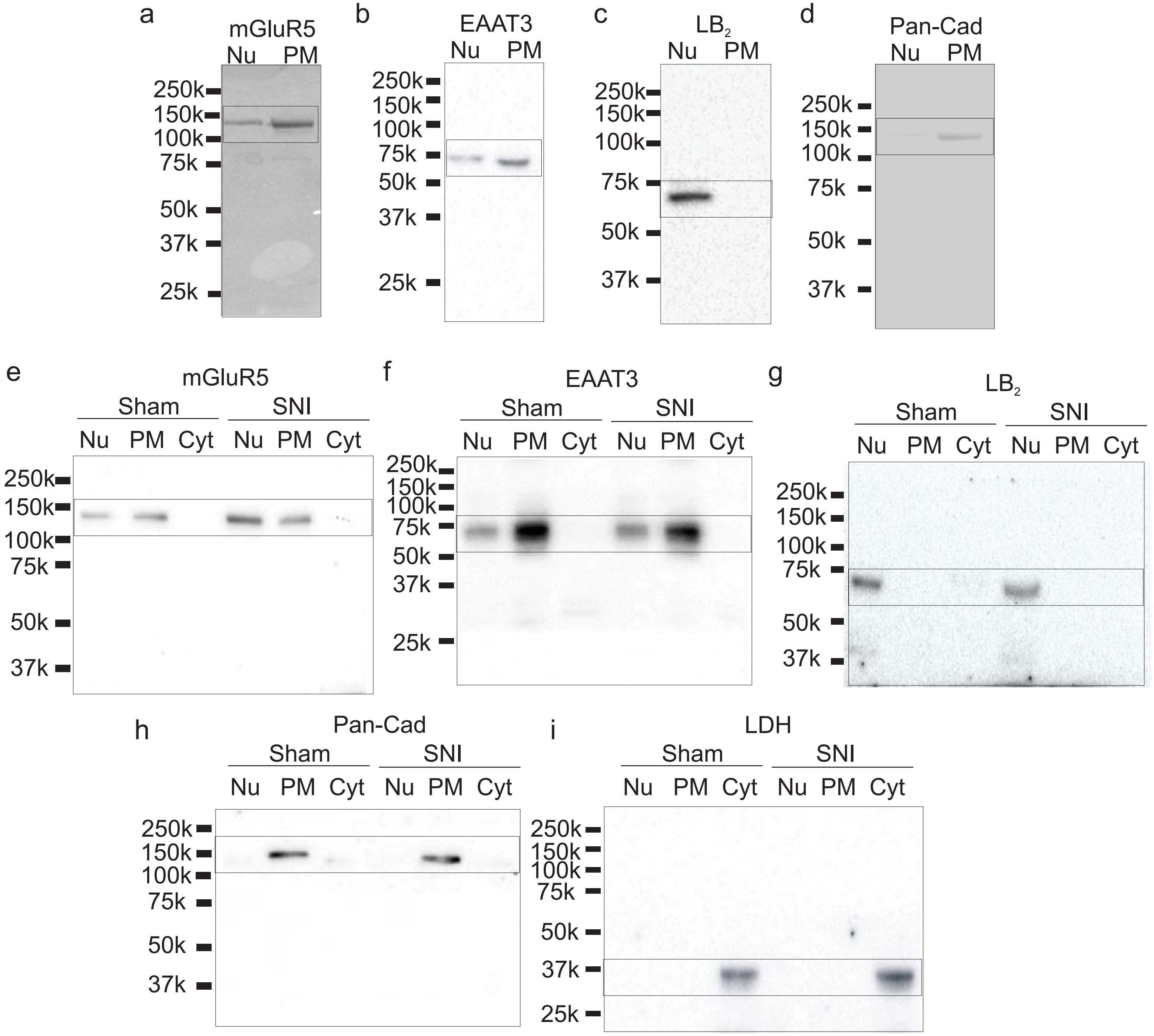

Supplementary Figure 7: Full length western blots for mGluR5, EAAT3 and cellular markers. Uncropped immunoblots of **a**) mGluR5, **b**) EAAT3, **c**) LB_2 (Lamin B_2)**,** **d**) Pan-Cad (Pan-cadherin) comprising panels in Figure 1d**,** and uncropped immunoblots of **e**) mGluR5, **f**) EAAT3, **g**) LB_2 **h**) Pan-Cad and **i**) LDH (lactate dehydrogenase) comprising panels in Figure 3d. Molecular weights are indicated in the scale on the left (SNI, spared nerve injury; Nu, nuclear; PM, plasma membrane; Cyt, cytosolic).

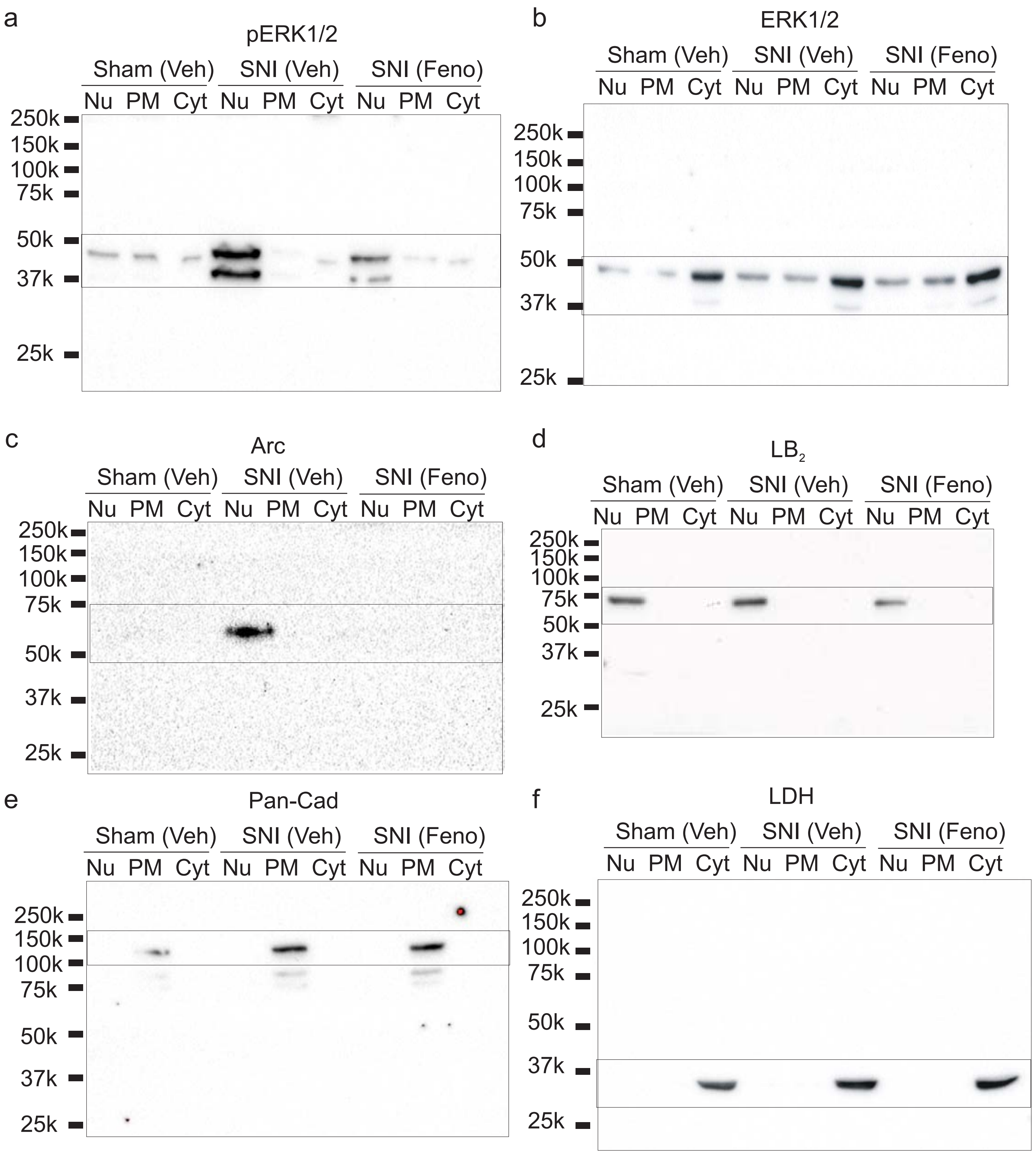

Supplementary Figure 8: Full length western blots for pERK1/2, ERK1/2 and Arc/Arg3.1 (Arc) and cellular markers. Uncropped immunoblots of **a**) pERK1/2, **b**) ERK1/2, **c**) Arc, **d**) LB_2 (lamin B_2), **e**) Pan-Cad (Pan-cadherin), and **f**) LDH (lactate dehydrogenase) comprising panels in Figure 4a. Molecular weights are indicated in the scale on the left (SNI, spared nerve injury; Veh, vehicle; Feno, fenobam; Nu, nuclear; PM, plasma membrane; Cyt, cytosolic).
